# Telehealth in primary health care: a study of activities and time spent by professionals*

**DOI:** 10.1590/1518-8345.7255.4500

**Published:** 2025-03-14

**Authors:** Debora Bernardo, Daiana Bonfim, Leticia Yamawaka de Almeida, Andrea Liliana Vesga-Varela, Natalia Martins Bonassi, Lorrayne Belotti

**Affiliations:** 1 Hospital Israelita Albert Einstein, Centro de Estudos, Pesquisa e Prática em Atenção Primária à Saúde e Redes, São Paulo, SP, Brazil; 2 Scholarship holder at the Associação Brasileira de Gestão em Projetos do Hospital Israelita Albert Einstein, São Paulo, SP, Brazil; 3 Hospital Israelita Albert Einstein, Saúde Digital, São Paulo, SP, Brazil

**Keywords:** Nursing, Primary Health Care, Telemedicine, Time and Motion Studies, Health Services, Professional Practice

## Abstract

to describe the telehealth activities carried out by professionals in Primary Health Care (PHC).

descriptive observational study, using time-motion methodology. The sample consisted of 31 Family Health teams, 14 Oral Health teams and two multidisciplinary teams working in PHC. An adapted workload instrument was used to collect the data. A descriptive analysis of the data was carried out considering the total time, average time and percentage of activities according to professional category and telehealth modality.

632 telehealth activities were observed, representing 2.67% of all activities. The most used modality was voice calls (60.28%). The average nursing consultation time was 4.86 minutes and 6.17 minutes for medical consultations. The greatest number of telehealth activities and time spent in PHC was carried out by the nursing team.

PHC professionals carry out a variety of telehealth activities, predominantly by voice connection, with the nursing team being the most expressive. In addition, the study shows the time dedicated and the distribution of activities, supporting discussions on the planning and sizing of the digital health workforce.

## Highlights

**(1)** Telehealth practices in PHC take place via voice connection. **(2)** The nursing team carries out the highest volume of telehealth activities in PHC. **(3)** Consultations accounted for the largest share of time and frequency of telehealth activities in PHC.

## Introduction

The rapid changes in Information and Communication Technologies (ICTs) have increased the possibility of their use, as well as their importance for access to healthcare. These changes have not only influenced traditional methods of health care and service delivery, but have also given rise to telehealth, a tool that can significantly improve access to health services^(1)^. This advance can mean an agile response to contemporary challenges, providing a more efficient and inclusive approach to the provision of healthcare^(2)^.

In this context, telehealth is an important strategy in the remote provision of health services, promoting instant communication between the individual and the health professional through electronic audio and video resources. This strategy aims to mitigate geographical obstacles, enabling collaboration between health professionals and the use of electronic devices for intervention or telediagnosis^(3-4)^.

Although telehealth is not a new concept and has been used in many healthcare organizations and systems, the COVID-19 pandemic has intensified its use in different healthcare settings^(5)^. Its incorporation into primary care, for example, has gained relevance due to the need to monitor people with respiratory symptoms and ensure continuity of care for the successful management of chronic diseases^(6)^.

In Brazil, telehealth, regulated by Law 14.510 of December 27, 2022^(7)^, aims to expand the population’s access to the Unified Health System (SUS), complementing face-to-face actions. It also covers activities such as monitoring, diagnosis, treatment and epidemiological surveillance to provide care, prevention, promotion, education, research, and health management^(8)^.

Primary Health Care (PHC) provides benefits such as continuous access to health services in remote areas, promoting people’s self-management and empowerment, reducing costs, and minimizing the need to travel for health care^(9)^. These elements contribute to achieving equity in access to healthcare, especially in remote and rural areas. It also facilitates communication between users, family caregivers, and health professionals, promoting the sharing of critical information^(1)^.

A study in Brazil examined the implementation of the National Telehealth Program Brazil Networks in PHC, highlighting teleeducation as the modality most frequently adopted by health teams. In addition, it was found that the highest rates of use occurred in the South and Southeast regions of the country, in municipalities with up to 30,000 inhabitants, with at least one physician present and a telephone available in the establishment. Structural aspects, such as the presence of the internet and multifunctional rooms, did not significantly impact the adoption of these technologies^(10)^.

However, factors such as the availability of a video-enabled device, a reasonably strong internet connection and digital literacy can be considered barriers to some people signing up for this type of care^(11)^. Furthermore, communicating about symptoms can be challenging for health service users, requiring ongoing follow-up to avoid possible harm caused by undiagnosed conditions^(12)^. Additionally, a study that examined the perspective of PHC professionals and managers about a telehealth program in a Brazilian capital identified challenges that included problems in using the system, issues related to infrastructure, practical difficulties in implementing the program, obstacles in integrating it into the organizational culture and a lack of management support^(13)^.

Although the literature has addressed the incorporation of telehealth into PHC, especially in the context of the COVID-19 pandemic, there is a significant gap in the detailed description of the specific activities carried out by health professionals, the extent to which telehealth takes up the work time of PHC professionals and the average time spent on each of them. A previous study focused on the implementation of telehealth programs and the structural and technological challenges faced but needed to go into a detailed analysis of telehealth practices in the daily lives of health teams, focusing on planning and optimizing the digital health workforce^(10)^.

Given this scenario, it is essential to identify the telehealth activities carried out by health professionals in PHC in the Brazilian context, situating this practice within the scope of the teams’ work process and promoting the expansion of the debate on workforce planning in the face of the introduction of new tools in everyday health care. This study, therefore, aims to describe the telehealth activities carried out by professionals in Primary Health Care.

## Method

### Study design

A descriptive observational study using the time-motion technique, which is a continuous observation technique that makes it possible to collect data in detail in order to determine the activities and the amount of time that professionals dedicate to carrying them out during the working day^(14)^.

### Setting

The study was carried out in five Primary Care Center (PCC) located in the southern region of a large municipality in the state of São Paulo (SP), Brazil. These services, located in vulnerable territories^(15)^, have approximately 115,000 registered people, operate from Monday to Friday (from 7am to 7pm) and have 31 Family Health teams, 14 Oral Health teams and two multidisciplinary teams (eMulti). All the health teams have a smartphone model cell phone made available by the managing institution to provide telehealth during the work routine since the beginning of the COVID-19 pandemic.

### Population

The study population was made up of health professionals with higher and secondary education, who met the inclusion criteria and were invited to take part in the study, linked to the aforementioned teams and distributed in the following categories: 12 dental surgeons, five oral health technicians, three oral health assistants, 11 physicians, 21 nurses, 15 nursing assistants, 18 community health workers (CHW) and eMulti professionals (one nutritionist, one social worker, one pharmacist, one environmental promotion workers two psychologists, one speech therapist, one physiotherapist and one occupational therapist).

To recruit participants, the coordinators of each PCC were contacted first. With the agreement of the local coordinators and in accordance with the health protocols for the period, the researchers held a group meeting with the health professionals in each of the services to present the research and formalize the invitation to the health professionals. On this occasion, the ethical aspects of the research were put into context and, at this point, the professionals were invited to access an electronic form using a QR code to read the Informed Consent Form. In addition, when appropriate, professionals who did not attend these meetings were contacted individually to present the proposal and formalize the invitation to take part in the study.

Those who showed interest and agreed to take part in the study were asked to fill in a questionnaire with items related to their sociodemographic and work profile. Secondly, an agreement was reached with the service coordinators and health professionals to align the data collection period and define a schedule for each unit.

### Data collection instruments and variables

The workload measurement tool^(16)^ was adapted and used to guide the recording of activities carried out by health professionals. In this study, a list of 41 activities were considered, organized into direct and indirect care interventions, associated activities and waiting time, as described below:

Direct care: activities carried out in the presence of the user/family/community. Examples: assistance with exams/procedures; care to spontaneous demand; consultations, immunization/vaccination checks; guidance on the health system and home visits^(16)^.

Indirect care: activities carried out without the presence of the user/family/community, but for their benefit. Examples: educational actions by health workers; student support; performance evaluation; supply control; development of care processes and protocols; development of community health; documentation; risk identification; organization of the work process; scientific research; referral and counter-referral; administrative meeting; meeting to evaluate multi-professional care; safety supervision; exchange of information on health care and/or health service; health surveillance^(16)^.

Associated activity: these are activities that can be carried out by other professional categories, but which the health professional takes on^(16)^. Examples: looking for material; looking for medical records; cleaning the unit, looking for another professional in the unit; looking for a room, etc.

Waiting time: when the professional is at their workstation available for care, waiting for the user and/or professional^(16)^.

It should be noted that, in order to identify telehealth activities during the observation, an item was included in the instrument to record the format in which the activity was carried out (face-to-face or remote). For remote activities, the following options were mapped as technological modalities: voice call, video call or instant text or voice messages carried out using a communication application *(WhatsApp Business)*.

### Data collection

Data collection was carried out in two stages to meet the health recommendations for the COVID-19 pandemic period, from March to August 2021, and from March to August 2022. This stage was conducted by a team of 10 higher education professionals, consisting of five nurses, two obstetricians, two dental surgeons and a psychologist, duly trained (120 theoretical-practical hours) by the researcher responsible for developing the project, to carry out direct and continuous observation of the work routine of health professionals, using the time motion technique^(17)^. Given the need for a designated observer for each professional, one researcher was assigned the role of observer to follow a specific professional throughout all the activities carried out in a typical working week.

The observation, without interaction or intervention, took place over 179 days and began when the health professional arrived at the UBS and ended at the end of their work shift, which varied between six and eight hours a day, depending on the professional category being investigated. The data was recorded simultaneously with the observation on a portable electronic device (tablet) used exclusively for the research and stored on Research Electronic Data Capture (REDCap)^(18-19)^, a data collection and management platform.

The scope of this study included activities carried out via voice calls, video calls or communication applications by the health professionals linked to the PCC that were part of the observations.

### Data processing and analysis

A descriptive analysis of the data was carried out using Excel software, taking into account the total time, average time and proportion of activities, considering the different professional categories and telehealth service modalities (voice call, video call or instant text or voice messages). Absolute and relative frequency tables were constructed and the data presented in graphs. For nurses, a detailed description of the consultation activity was made, considering the volume of observations made by these professionals.

### Ethical aspects

The study was approved by the Research Ethics Committee of the responsible institution (CAAE: 23388819.9.0000.0071) on November 28, 2019, and by the health institution of the municipality studied (CAAE: 23388819.9.3001.0086) on January 5, 2020.

## Results

A total of 82 (87.00%) professionals carried out at least one telehealth activity: 10 dental surgeons, five oral health technicians, one oral health assistant, nine physicians, 21 nurses, 12 nursing assistants, 16 CHWs, and eight eMulti professionals (one nutritionist, one social worker, one pharmacist, one environmental promotion worker, two psychologists, one speech therapist, and one physiotherapist).

During the period in which these professionals’ working hours were monitored, 632 telehealth activities were observed, which corresponded to almost 3.00% of the total activities carried out. In terms of the total volume of telehealth activities among all the activities observed, the eMulti team was the one that had most of its activities carried out in the telehealth format (7.53%), followed by nurses (3.93%). About telehealth modalities, 60.28% of the activities were conducted via voice calls, 32.28% via an app and 7.44% via video calls (Table 1).

**Table 1 -:** Distribution of activities observed according to the face-to-face or telehealth format carried out by professionals in PHC. São Paulo, SP, Brazil, 2021-2022

**Professional categories**	**Face-to-face**		**Telehealth**							
			**Total**		**Voice**		**Video**		App*	
	n	%	n	%	n	%	n	%	n	%
Nursing Assistant	4506	99.10	41	0.90	18	43.90	11	26.83	12	29.27
Nurse	6276	96.07	247	3.93	161	65.18	17	6.89	69	27.93
Community Health Worker	4245	96.94	130	3.06	69	53.08	7	5.38	54	41.54
Physician	2810	96.91	87	3.09	70	80.45	2	2.30	15	17.25
Dental Surgeon	1829	98.26	32	1.74	15	46.88	7	21.87	10	31.25
Oral Health Assistant	597	99.00	6	1.00	-	-	-	-	6	100
Oral Health Technician	789	97.98	16	2.02	16	100	-	-	-	-
eMulti	969	92.47	73	7.53	32	43.83	3	4.11	38	52.06
**Total**	**22021**	**97.14**	**632**	**2.86**	**381**	**60.28**	**47**	**7.44**	**204**	**32.28**

* App = WhatsApp Business (text and voice messages)

**Figure 1 -:**
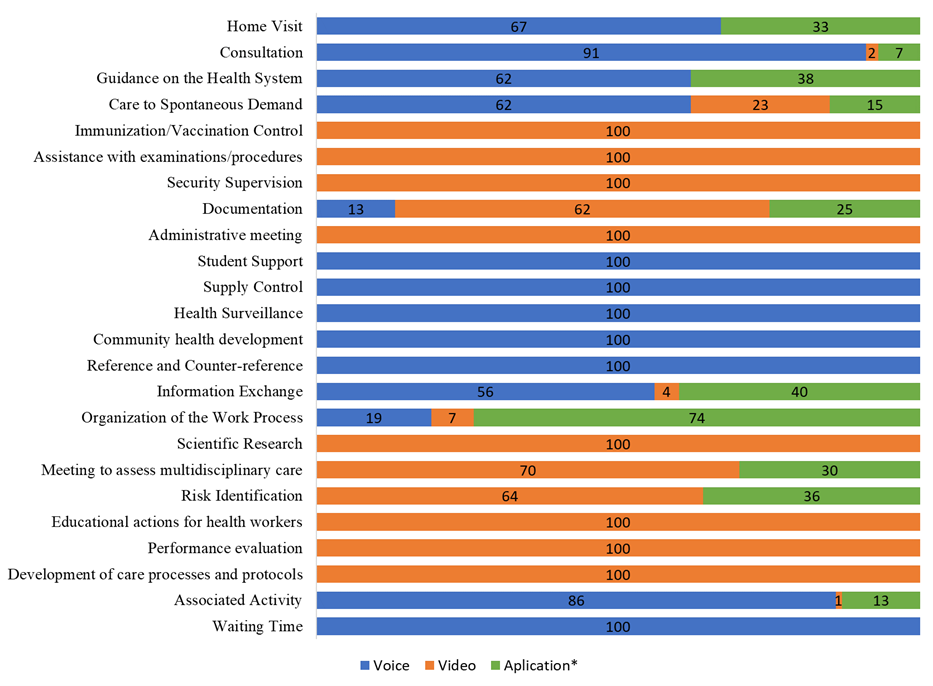
Percentage of total time spent on telehealth activities according to the modality used (voice call, video call and messaging app). São Paulo, SP, Brazil, 2021-2022

Figure 1 shows the distribution of telehealth activities carried out by the professionals taking part in the study, classified according to the type of service (voice, video and application). Notably, six activities were carried out exclusively by voice, while eight took place exclusively by video. With regard to home visits carried out remotely, 67.00% took place by voice, while 33.00% used the communication app *(WhatsApp Business)*. In the context of consultations, 91.00% were conducted by voice, 2.00% by video and 7.00% by app.

In total, 24 categories of telehealth activities integrated into professionals’ routines were identified, divided into six direct care activities and eighteen indirect care activities. Overall, the most frequently performed remote activities were information exchange (n=155) and consultations (n=153). In terms of time parameters, there were 4,669 minutes dedicated to telehealth activities, distributed between 1,355 minutes of direct care, 2,988 minutes of indirect care, 31 minutes of waiting and 295 minutes of associated activities (Table 2).

*App = WhatsApp Business (text and voice messages)

Consultation stood out as the telehealth activity with the most time spent by professionals, totaling 863 minutes observed among all professionals, most frequently conducted by physicians (46.47%) and then by nurses (43.92%). The average time taken by nurses to conduct a consultation remotely was 4.86 minutes and 6.17 minutes for medical consultations.

Telehealth activities related to Health Surveillance totaled 552 minutes, of which nurses conducted 62.14%. In addition, among the telehealth activities carried out by the participants (n=632), professionals from the nursing team (nursing assistants and nurses) stood out, totaling 288 actions and representing approximately 46.00% of the total telehealth activities observed. The activity of exchanging information about health care and/or health services had 446 minutes of telehealth activity, in which nurses and eMulti were the ones who carried out this type of activity the most, the majority of which was via voice call (Table 2).

**Table 2 -:** Frequency, total time in minutes, average time in minutes and percentage of time of telehealth activities carried out by professionals. São Paulo, SP, Brazil, 2021-2022

Activities	N Total	TT*	Nursing assistant				Nurse			Community health worker			Physician			Dental Surgeon			eMulti	
			n	MT†	% of TT*	n	TM†	% of TT*	n	MT†	% of TT*	n	MT†	% of TT*	n	MT†	% of TT*	n	MT†	% of TT*
Assistance with exams/procedures	1	4.00	1	4.00‡	100	-	-	-	-	-	-	-	-	-	-	-	-	-	-	-
Care to demand assistance	6	26.00	1	4.00‡	15.38	-	-	-	-	-	-	1	6.00‡	23.07	-	-	-	4	4.00	61.53
Consultation	153	863.00	-	-	-	78	4.86	43.92	-	-	-	65	6.17	46.47	4	8.00	3.70	6	8.50	5.91
Immunization/Vaccination Control	5	40.00	2	16.50	82.50	3	2.33	17.50	-	-	-	-	-	-	-	-	-	-	-	-
Guidance on the health system	79	290.00	3.80	6.55	30	3.80	39.31	41	3.59	50.69	-	-	-	-	-	-	-	1	3.00‡	1.03
Home visits	43	132.00	-	-	-	-	-	-	43	3.07	100	-	-	-	-	-	-	-	-	-
Educational activities for health workers	6	501.00	-	-	-	2	65.50	26.14	1	69.00‡	13.77	-	-	-	2	103.00	41.11	1	95.00‡	18.96
Student support	2	8.00	-	-	-	2	4.00	100	-	-	-	-	-	-	-	-	-	-	-	-
Performance evaluation	1	4.00	1	4.00‡	100	-	-	-	-	-	-	-	-	-	-	-	-	-	-	-
Supply control	3	15.00	3	5.00	100	-	-	-	-	-	-	-	-	-	-	-	-	-	-	-
Development of care processes and protocols	2	153.00	-	-	-	1	63.00‡	41.18	-	-	-	1	6.00‡	23.07	-	1	90.00‡	58.82	-	-
Community health development	1	10.00	-	-	-	-	-	-	-	-	-	-	-	-	-	-	-	1	10.00‡	100
Documentation	11	172.00	1	9.00‡	5.23	1	5.00‡	2.90	5	23.80	69.18	2	8.50	9.88	-	-	-	2	11.00	12.79
Risk identification	2	126.00	2	63.00	100	-	-	-	-	-	-	-	-	-	-	-	-	-	-	-
Organization of the work process	84	351.00	4	3.25	3.70	30	3.37	28.77	14	3.93	15.67	10	3.30	9.40	11	5.00	15.67	9	2.67	6.83
Scientific Research	3	184.00	-	-	-	-	-	-	-	-	-	-	-	-	3	61.33	100	-	-	-
Referral and Counter-referral	2	22.00	-	-	-	-	-	-	-	-	-	-	-	-	1	11.00‡	50	1	11.00‡	50
Administrative meeting	5	257.00	-	-	-	3	45.33	52.92		-	-	-	-	-	1	61.00‡	23.74	1	60.00‡	23.34
Multiprofessional care assessment meeting	5	184.00	-	-	-	3	21.33	34.78	1	64.00‡	34.78	-	-	-	-	-	-	1	56.00‡	30.44
Supervision Safety	1	3.00	1	3.00‡	100	-	-	-	-	-	-	-	-	-	-	-	-	-	-	-
Exchange of information on health care and/or health service	155	446.00	12	2.17	5.83	72	2.18	35.20	13	3.46	10.09	4	1.00	0.89	4	5.24	4.70	43	4.00	38.56
Health Surveillance	25	552.00	4	34.50	25.00	14	24.50	62.14	1	14‡	2.54	1	15.00‡	2.72	3	9.66	1.63	2	6.50	2.35
Waiting time	7	31.00	-	-	-	2	4.00	25.80	1	11‡	35.48	3	1.67	16.13	-	-	-	1	7.00‡	22.58
Associated Activity	30	295.00	4	2.75	3.73	6	5.33	10.85	10	3.70	12.54	1	2.00‡	0.68	2	3.50	2.37	-	-	-

*Total time in minutes; ^†^Average time in minutes; ^‡^When the activity was carried out only once, the total time was used

Table 3 describes in detail the consultation activities conducted by the nurses, categorized according to type. Among the types of consultations, those related to follow-up and hypertension/diabetes stood out, accounting for 22.70% and 17.73% respectively. As for the location, the physician’s office was the most frequent, accounting for 66.23% of consultations, while the action most observed during consultations was note-taking, accounting for 44.51%, followed by scheduling, which accounted for 15.09%. It can be seen that, in general, the majority of remote consultations were conducted by voice.

**Table 3 -:** Frequency and percentage of consultation activities in the telehealth modality carried out by nurses. São Paulo, SP, Brazil, 2021-2022

	**n (%)**	**Modality**		
		**Voice**	**Video**	App*
		n (%)	n (%)	n (%)
**Type of consultation** ^†^				
First appointment	7 (4.96)	7 (100.00)	-	-
Follow-up	32 (22.70)	27 (84.38)	1 (3.12)	4 (12.50)
Childcare	1 (0.71)	1 (100.00)	-	-
Women’s health	5 (3.55)	5 (100.00)	-	-
Hypertension and Diabetes Mellitus	25 (17.73)	25 (100.00)	-	-
Mental health	1 (0.71)	1 (100.00)	-	-
Test delivery	15 (10.64)	14 (93.33)	-	1 (6.70)
Others	55 (39.01)	55 (100.00)	-	-
**Place of consultation**				
Consulting room	51 (66.23)	39 (76.47)	1 (1.96)	11 (21.57)
Procedure room	4 (5.19)	4 (100.00)	-	-
Regulation room	1 (1.30)	1 (100.00)	-	-
Other rooms	21 (27.27)	21 (100.00)	-	-
**Actions taken within the consultation** ^†^				
Anamnesis	23 (21.7)	22 (95.65)	1 (4.35)	-
Physical examination	1 (0.94)	-	1 (100)	-
Prescription of medication	3 (2.83)	2 (66.67)	-	1 (3.33)
Requesting tests	3 (2.83)	2 (66.67)	1 (3.33)	-
Matriculation with eMulti ^‡^	1 (0.94)	1 (100.00)	-	-
Referral to specialist	4 (3.77)	4 (100.00)	-	-
Referral to eMulti ^‡^	2 (1.89)	2 (100.00)	-	-
Appointment scheduling	16 (15.09)	14 (87.5)	-	2 (12.50)
Annotation	44 (41.51)	43 (97.73)	1 (2.27)	-
Prescription exchange	8 (7.55)	7 (87.50)	-	1 (12.50)
Prevention activity	1 (0.94)	1 (100.00)	-	-

*App = WhatsApp Business (text and voice messages); ^†^Variables with more than one answer option; ^‡^Multidisciplinary team

## Discussion

This study provided a snapshot of the practice of telehealth in PHC in a Brazilian municipality. The professional categories that had most of their activities carried out in the telehealth format were the eMulti and nursing teams. However, the greatest contribution in terms of telehealth volume was made by the nursing team.

With regard to the total time dedicated to telehealth work, although professionals carry out various remote activities related to management, education, research and care, consultations accounted for the largest share of time, as well as high frequency, followed by health surveillance actions, continuing education and exchange of information between professionals. It should also be noted that the most common form of telehealth in PHC was through voice calls.

Evidence has shown that telephone and videoconference consultations are as effective as face-to-face consultations in improving clinical outcomes in adults attending PHC services^(2)^. However, although the transition from face-to-face consultations to telehealth has been intense during the pandemic, there are concerns about its use, as a study suggests potential racial, geographic and other disparities in access to care^(20)^.

Telehealth is a powerful tool for expanding access to consultations with PHC professionals. When incorporated, it seeks to ensure continuous monitoring and facilitate access for people who have difficulty getting to the health service, whether due to physical or organizational barriers, such as opening hours. Therefore, implementing of telehealth activities for PHC must incorporate skills and processes related to digital health equity^(21)^.

The results showed that nursing consultations are among the telehealth practices that spend the most time on nurses in PHC, but they are still mostly carried out by voice. In this way, one study pointed to the importance of advancing and instrumentalizing the incorporation of the nursing process into the remote approach^(22)^.

A study carried out in Canada showed that nurses considered the pandemic to be the main catalyst for the implementation of teleconsultations, but that after the peak of the pandemic, face-to-face consultations gradually replaced teleconsultations, suggesting concrete solutions to encourage their implementation after the pandemic, such as: integrating teleconsultations into existing workflows, so that they can provide an advantage over face-to-face consultations; adopting policies and protocols to facilitate the integration of teleconsultation modalities into care, as well as directing users to the most appropriate professional to meet their needs; and the continuous and permanent education and training of nurses, adapted to needs and with technology that is simple to use^(23)^.

The proposal for Telehealth Services to support Continuing Education has been discussed over the years in Brazil^(24-25)^, and has the potential to increase professionals’ access to training and development activities. Professional categories such as dental surgeons and nurses stand out in terms of the amount of time spent, but continuing education via telehealth is still not very significant in the routine practices of nursing assistants, demonstrating a potential opportunity for the insertion of telehealth in all professional categories, and the need to develop this competence, so that the PHC team can use telehealth as a powerful tool for qualifying the work process, as well as strengthening the coordination of care, access and links between users, families and the community.

It is noteworthy that the exchange of information was the activity most frequently conducted remotely by all the professionals, representing one of the most frequent practices in the telehealth modality adopted by the eMulti team. Due to its short duration, this activity ranked among the top four when analyzing the amount of time dedicated. ICTs offer various possibilities for interactivity and exchange between professionals, characterizing them as an important support tool for shared decision-making^(26)^.

Actions made possible by remote care technologies, such as health surveillance, were incorporated into the activities of all the professionals observed, but nursing assistants and nurses were the ones who spent the most time working. In that regard, Canadian nurses report that before the pandemic, their telehealth activities in PHC were more focused on monitoring test results and some chronic diseases by telephone^(23)^, characteristics also found in the Brazilian reality. However, they intensified with the pandemic period, such as monitoring cases and test results and the rapid adaptation made by the teams with the incorporation of ICTs^(27)^. Research carried out in the state of Bahia has demonstrated the importance of telehealth and digital health technologies as central to monitoring the spread of other emerging and re-emerging diseases^(28)^.

In addition, although the link between climate and health has been researched in recent years and has demonstrated the impacts of climate change on health^(29)^, few investigations debate how these ongoing climate changes can impact the activities and work of health professionals in PHC, such as CHWs. Thus, telehealth activities for these professionals could be key tools for enabling home visits in the face of heat waves, cold waves, floods, droughts, and other extreme climatic events that can, directly and indirectly impact health.

In this study, it was observed that the voice modality was the most frequently used during telehealth activities. A previous study comparing two telehealth methods in PHC (voice and video) indicated that, in terms of duration, content and quality of the consultation, both are similar^(30-31)^. However, videoconferencing can offer an advantage, as it allows health professionals to see people, helping to establish a more solid relationship and improving communication between both parties^(9)^. However, technical problems and inequalities in access to and use of digital devices with cameras are more common compared to voice-only calls^(32)^, as well as the preference of the population, as demonstrated by a study in Australia which found that although legislators preferred video consultations, the majority of telehealth consultations were carried out by telephone, suggesting that there are still barriers to video consultations^(33)^.

Thus, the adequacy of telehealth services, considering video or voice connection, is complex and linked to different factors, such as people’s preferences, digital access, specific health conditions and the needs of team members. In a study conducted in the United States, health professionals from a primary care team highlighted the relevance of user preferences when adapting telehealth services. Some people opted for virtual meetings due to the convenience in terms of cost and travel time, while others faced challenges such as limited internet access or the absence of a telephone handset^(9)^.

From the perspective of health service users, some advantages of telehealth services stand out, such as easier access to health care, reduced waiting times and the provision of health care without the risk of exposure to COVID-19, especially when transmission and infection rates were high^(34)^. However, there are challenges to be overcome, both for people who use health services and for health professionals. For people, these challenges include difficulties in accessing and using technologies, concerns about engaging with users’ expectations and providing virtual care for rural, older adult or cognitively impaired populations. For professionals, they involve the impossibility of carrying out physical examinations, the limitation of personal contact and touch, diagnostic difficulties in the absence of physical examinations, technological restrictions, maintaining the therapeutic relationship and ensuring parity of payment between face-to-face and virtual visits^(9)^.

In addition, considering that the telehealth service in PHC was strengthened with the COVID-19 pandemic through experiences of readapting the services offered, seeking a new path for the continuity of care for the population, with actions that ranged from readjustments in infrastructure and care flows to telemedicine as an alternative modality of care and monitoring of cases through applications and via telephone^(35)^. However, although the experiences strengthened the opportunities for consolidating telehealth and improving the SUS, after this period, debates questioned the permanence of telehealth, given the return of face-to-face activities, growing demand in PHC services and changes in the work process, which were not accompanied by discussions on the new sizing of the PHC workforce.

A study has shown that little is known about the real or ideal state of telehealth workforce needs in low- and middle-income countries^(36)^. In addition, few studies present time parameters, such as those found in this study, which can support the planning and sizing of the health workforce in a scientific technical way.

From this perspective, a research carried out in Ghana used the Workload Indicators of Staffing Need methodology to assess the sufficiency of the digital health workforce and estimate staff shortages/excesses and workload pressures, as little is known about the necessary mix and size of the workforce that would be required to effectively manage a digital health system in a health service, thus finding inadequate workforce numbers and an unbalanced mix of skills^(36)^.

Finally, this study has some limitations that should be considered. Firstly, data collection took place during the COVID-19 pandemic, which may have influenced the activities carried out by the healthcare team, reflecting a temporary adaptation to the pandemic circumstances. In addition, the methodology adopted, based on observation, may not have fully captured activities that were carried out quickly or that involved multiple simultaneous tasks. These aspects may have led to underestimates or omissions in the recording of activities, limiting the generalizability of the results.

Despite the limitations, the results of this study have important implications for the practice of health services. The use of direct observation by external observers, using the time motion technique, provided more accurate and reliable data than subjective methods, such as self-reporting, on the activities carried out by health professionals. By providing a detailed and unprecedented overview of the time dedicated to telehealth, especially by the nursing category, the study contributes to the scientific debate on the incorporation of this technology into PHC. Telehealth, a technology with great potential for expanding access to PHC, is proving to be an essential tool for qualifying work and strengthening the coordination of care in the SUS.

## Conclusion

In summary, the telehealth activities carried out by PHC professionals are diverse, integrating activities related to management, education, research and care practices, distributed in different proportions over the working day. The activities that concentrated the most time in the working day stand out, such as consultations, health surveillance, educational activities for health workers and the exchange of information about health care and/or health services, most of which were carried out via voice calls. In addition, the study presents average time patterns for telehealth activities, supporting the discussion of planning and sizing the digital health workforce in the context of PHC.
