# Supplementary material for: Telehealth in primary health care: a study of activities and time spent by professionals
Source: Rev Lat Am Enfermagem. 2025 Mar 14;33:e4500. doi: 10.1590/1518-8345.7255.4500 (PMC11912818; doi:10.1590/1518-8345.7255.4500)
Supplement: Translation

# Translation: Telessaúde na atenção primária à saúde: um estudo das atividades e do tempo despendido pelos profissionais*

**DOI:** 10.1590/1518-8345.7255.4501

**Authors:** Debora Bernardo, Daiana Bonfim, Leticia Yamawaka de Almeida, Andrea Liliana Vesga-Varela, Natalia Martins Bonassi, Lorrayne Belotti

**Affiliations:** 1 Hospital Israelita Albert Einstein, Centro de Estudos, Pesquisa e Prática em Atenção Primária à Saúde e Redes, São Paulo, SP, Brasil; 2 Bolsista da Associação Brasileira de Gestão em Projetos do Hospital Israelita Albert Einstein, São Paulo, SP, Brasil; 3 Hospital Israelita Albert Einstein, Saúde Digital, São Paulo, SP, Brasil

**Keywords:** Enfermagem, Atenção Primária à Saúde, Serviços de Telessaúde, Estudos de Tempo e Movimento, Serviços de Saúde, Prática Profissional

## Abstract

descrever as atividades de telessaúde realizadas pelos profissionais na Atenção Primária à Saúde (APS).

estudo observacional descritivo, com utilização da metodologia *time-motion*. A amostra foi composta por 31 equipes de Saúde da Família, 14 equipes de Saúde bucal e duas equipes multidisciplinares que atuam na APS. Para a coleta de dados, utilizou-se um instrumento de carga de trabalho adaptado. Foi realizada a análise descritiva dos dados considerando o tempo total, tempo médio e porcentagem das atividades conforme a categoria profissional e a modalidade de telessaúde.

foram observadas 632 atividades de telessaúde, representando 2,67% do total de atividades. A modalidade mais utilizada foi a ligação de voz (60,28%). O tempo médio da consulta de enfermagem foi de 4,86 minutos e 6,17 minutos para consulta médica. O maior número de atividades e de tempo despendido de telessaúde na APS foi realizada pela equipe de enfermagem.

os profissionais da APS realizam diversas atividades de telessaúde, predominantemente por ligação de voz, com expressividade da equipe de enfermagem. Além disso, o estudo apresenta o tempo dedicado e a distribuição das atividades, subsidiando discussões sobre o planejamento e dimensionamento da força de trabalho em saúde digital.

## Destaques

**(1)** As práticas de telessaúde na APS ocorrem por meio de ligação de voz. **(2)** A equipe de enfermagem realiza o maior volume de atividades de telessaúde na APS. **(3)** Consultas representaram a maior parcela de tempo e frequência de atividades de telessaúde na APS.

## Introdução

As rápidas transformações nas tecnologias de telecomunicação (TICs) ampliaram a possibilidade do seu uso, bem como importância para o acesso à saúde. Essas mudanças não apenas influenciaram os métodos tradicionais de atendimento e prestação de serviços de saúde, mas também deram origem à telessaúde, uma ferramenta que pode aprimorar significativamente o acesso aos serviços de saúde^(1)^. Este avanço pode significar uma resposta ágil aos desafios contemporâneos, proporcionando uma abordagem mais eficiente e inclusiva na oferta de cuidados em saúde^(2)^.

Nesse contexto, a telessaúde se configura como uma importante estratégia na oferta remota de serviços de saúde, promovendo a comunicação instantânea entre o indivíduo e o profissional de saúde por meio de recursos eletrônicos de áudio e vídeo. Tal estratégia visa mitigar os obstáculos geográficos, possibilitando a colaboração entre profissionais de saúde e a utilização de dispositivos eletrônicos para intervenção ou telediagnóstico^(3-4)^.

Embora a telessaúde não seja um conceito novo e tenha sido utilizada em muitas organizações e sistemas de saúde, a pandemia de COVID-19 intensificou sua utilização em diferentes ambientes de cuidados de saúde^(5)^. A sua incorporação nos cuidados primários, por exemplo, ganhou relevância devido à necessidade de monitorar as pessoas com sintomas respiratórios e assegurar a continuidade dos cuidados para uma gestão bem-sucedida de doenças crônicas^(6)^.

No Brasil, a telessaúde - regulamentada pela Lei 14.510, de 27 de dezembro de 2022^(7)^ - tem como objetivo ampliar o acesso da população ao Sistema Único de Saúde (SUS), complementando as ações presenciais. Ainda, abrange atividades como acompanhamento, diagnóstico, tratamento e vigilância epidemiológica, visando fornecer assistência, prevenção, promoção, educação, pesquisa e gestão em saúde^(8)^.

No âmbito da Atenção Primária a Saúde (APS), proporciona benefícios, como o acesso contínuo a serviços de saúde em áreas remotas; promoção da autogestão e capacitação das pessoas; redução de custos; e minimização da necessidade de deslocamentos para cuidados em saúde^(9)^. Esses elementos contribuem para alcançar a equidade no acesso à saúde, especialmente em áreas remotas e rurais. Além disso, facilita a comunicação entre pessoas usuárias, cuidadores familiares e profissionais de saúde, promovendo o compartilhamento de informações críticas^(1)^.

Estudo realizado no Brasil, que examinou a implementação do Programa Nacional Telessaúde Brasil Redes na APS, destacou a teleducação como a modalidade mais frequentemente adotada pelas equipes de saúde. Adicionalmente, constatou-se que as maiores taxas de utilização ocorreram nas regiões Sul e Sudeste do país, em municípios com até 30 mil habitantes, com pelo menos um médico presente e um telefone disponível no estabelecimento. Aspectos estruturais, como a presença de internet e de salas multifuncionais, não tiveram um impacto significativo na adoção dessas tecnologias^(10)^.

Entretanto, fatores como disponibilização de um dispositivo habilitado para vídeo, uma conexão de internet razoavelmente forte e alfabetização digital podem ser considerados barreiras para a adesão de algumas pessoas para este tipo de atendimento^(11)^. Além disso, a comunicação sobre os sintomas pode ser desafiadora para as pessoas usuárias do serviço de saúde, exigindo acompanhamento contínuo para evitar possíveis danos causados por condições não diagnosticadas^(12)^. Adicionalmente, uma pesquisa que examinou a perspectiva de profissionais e gestores da APS acerca de um programa de telessaúde em uma capital brasileira identificou desafios que incluíam: problemas no uso do sistema, questões relacionadas à infraestrutura, dificuldades práticas na implementação do programa, entraves na integração à cultura organizacional e carência de suporte gerencial^(13)^.

Ainda que a literatura tenha abordado a incorporação da telessaúde na APS, principalmente no contexto da pandemia de COVID-19, há uma lacuna significativa quanto à descrição detalhada das atividades específicas realizadas pelos profissionais de saúde, o quanto a telessaúde ocupa do tempo de trabalho dos profissionais na APS e parâmetros de tempo médio despendido em cada uma delas. Um estudo realizado anteriormente focou na implementação de programas de telessaúde e nos desafios estruturais e tecnológicos enfrentados, todavia não avançaram em uma análise detalhada das práticas de telessaúde no cotidiano das equipes de saúde, com foco no planejamento e na otimização da força de trabalho em saúde digital^(10)^.

Diante desse cenário, torna-se essencial a identificação das atividades de telessaúde desempenhadas pelos profissionais de saúde na APS no contexto brasileiro, situando essa prática no âmbito do processo de trabalho das equipes e promovendo a ampliação do debate acerca do planejamento da força de trabalho frente à introdução de novas ferramentas no cotidiano do cuidado em saúde. Assim, o presente estudo tem como objetivo descrever as atividades de telessaúde realizadas pelos profissionais na Atenção Primária à Saúde.

## Método

### Delineamento do estudo

Estudo observacional descritivo, em que foi utilizada a técnica *time-motion*, que é uma técnica de observação contínua que possibilita a coleta de dados de maneira detalhada com o objetivo de determinar as atividades e o volume de tempo que os profissionais dedicam para sua realização no período da jornada de trabalho^(14)^.

### Cenário

O estudo foi desenvolvido em cinco Unidades Básicas de Saúde (UBS), localizadas na região sul de um município de grande porte do estado de São Paulo (SP), Brasil. Estes serviços, inseridos em territórios vulneráveis^(15)^, possuem aproximadamente 115 mil pessoas cadastradas, funcionam de segunda a sexta-feira (das 7h às 19h) e contam com a atuação de 31 equipes de Saúde da Família, 14 equipes de Saúde Bucal e duas equipes multidisciplinares (eMulti). Todas as equipes de saúde possuem um celular modelo *smartphone* que foi disponibilizado pela instituição gestora para oportunizar a telessaúde durante a rotina de trabalho desde o início do período de pandemia de COVID-19.

### População

A população do estudo foi constituída por profissionais de saúde com nível superior e médio de ensino, que atendiam aos critérios de inclusão e foram convidados a participar do estudo, vinculados às equipes supracitadas e distribuídos nas seguintes categorias: 12 cirurgiões-dentistas, cinco técnicos de saúde bucal, três auxiliares de saúde bucal, 11 médicos, 21 enfermeiros, 15 auxiliares de enfermagem, 18 agentes comunitários de saúde (ACS) e profissionais da eMulti (um nutricionista, um assistente social, um farmacêutico, um agente de promoção ambiental, dois psicólogos, um fonoaudiólogo, um fisioterapeuta e um terapeuta ocupacional).

Para o recrutamento dos participantes, inicialmente foi realizado o contato com a coordenação de cada UBS. Com a anuência dos coordenadores locais e de acordo com os protocolos sanitários do período, os pesquisadores conduziram um encontro em grupo com os profissionais de saúde em cada um dos serviços para a apresentação da pesquisa e a formalização do convite aos profissionais de saúde. Na ocasião, realizou-se a contextualização dos aspectos éticos em pesquisa e, neste momento, os profissionais eram convidados a acessar um formulário eletrônico por meio de *QR code* para leitura do Termo de Consentimento Livre e Esclarecido. Salienta-se ainda que, para os profissionais que por algum motivo não participaram destes encontros, foi realizado, quando oportuno, o contato de forma individual para apresentação da proposta e formalização do convite para participação no estudo.

Aqueles que manifestaram interesse e concordância em participar da pesquisa eram direcionados para o preenchimento de um questionário com itens relacionados ao perfil sociodemográfico e laboral. Em um segundo momento, foi realizada a pactuação com a coordenação dos serviços e com os profissionais de saúde para alinhamento do período de coleta de dados e definição de um cronograma para cada unidade.

### Instrumentos de coleta de dados e variáveis

O instrumento de medida da carga de trabalho^(16)^ foi adaptado e utilizado para nortear o registro das atividades realizadas pelos profissionais de saúde. No presente estudo, foram considerados um rol de 41 atividades, organizadas em intervenções de cuidado direto e indireto, atividades associada e tempo de espera, conforme descrito a seguir:

Cuidado direto: são atividades realizadas na presença do usuário/família/comunidade. Exemplos: assistência em exames/procedimentos; atendimento a demanda espontânea; consulta, controle de imunização/vacinação; orientação quanto ao sistema de saúde e visita domiciliar^(16)^.

Cuidado indireto: são atividades realizadas sem a presença do usuário/família/comunidade, mas em seu benefício. Exemplos: ações educativas dos trabalhadores de saúde; apoio ao estudante; avaliação de desempenho; controle de suprimentos; desenvolvimento de processos e protocolos de cuidado; desenvolvimento da saúde comunitária; documentação; identificação de risco; organização do processo de trabalho; pesquisa científica; referência e contrarreferência; reunião administrativa; reunião para avaliação dos cuidados multiprofissionais; supervisão segurança; troca de informações sobre cuidados de saúde e/ou serviço de saúde; vigilância em saúde^(16)^.

Atividade associada: são atividades que podem ser realizadas por outras categorias profissionais, mas que o profissional de saúde assume^(16)^. Exemplos: procurar material; procurar prontuário; limpeza da unidade, procurar por outro profissional pela unidade; procurar sala, outros.

Tempo de espera: quando o profissional está em seu posto de trabalho disponível para o atendimento, aguardando o usuário e/ou profissional^(16)^.

Destaca-se que, para identificação das atividades de telessaúde durante a observação, foi incluído um item no instrumento para o registro do formato que a atividade era desenvolvida (presencial ou remoto). Para as atividades remotas, foram mapeadas como modalidades tecnológicas as seguintes opções: ligação de voz, ligação de vídeo ou mensagens instantâneas de texto ou voz realizadas por meio do uso de aplicativo de comunicação *(WhatsApp Business)*.

### Coleta de dados

A coleta de dados foi realizada em dois momentos para atender às recomendações sanitárias do período de pandemia de COVID-19, nos períodos de março a agosto de 2021, bem como de março a agosto de 2022. Essa etapa foi conduzida por uma equipe de 10 profissionais de nível superior, bolsistas, composta por cinco enfermeiros, dois obstetrizes, dois cirurgiões-dentistas e um psicólogo, devidamente treinados (120 horas teórico-práticas) pelo pesquisador responsável pelo desenvolvimento do projeto, para realizar uma observação direta e contínua da rotina de trabalho dos profissionais de saúde, empregando a técnica *time motion* ^(17)^. Dada a necessidade de um observador designado para cada profissional, um pesquisador foi atribuído à função de observador, para acompanhar um profissional específico ao longo de todas as atividades realizadas em uma semana típica de trabalho.

A observação, sem interação ou intervenção, ocorreu por 179 dias. Ela iniciava-se com a chegada do profissional de saúde à UBS, encerrando-se ao final do turno de trabalho, que variava entre seis a oito horas diárias, dependendo da categoria profissional investigada. Os dados eram registrados simultaneamente à observação, em um dispositivo eletrônico portátil (*tablet*), que era utilizado exclusivamente para a pesquisa e armazenados na *Research Eletronic Data Capture* (REDCap)^(18-19)^, plataforma de coleta e gerenciamento dos dados.

No escopo deste estudo, foram incluídas as atividades efetuadas por meio de chamadas de voz, videochamadas ou aplicativos de comunicação pelos profissionais de saúde vinculados às UBS que integraram as observações.

### Tratamento e análise dos dados

Realizou-se uma análise descritiva dos dados utilizando o *software Excel*, contemplando o tempo total, o tempo médio e a proporção das atividades, considerando as diferentes categorias profissionais e as modalidades de atendimento de telessaúde (ligação de voz, ligação de vídeo ou mensagens instantâneas de texto ou voz). Em vista disso, foi realizada a construção de tabelas de frequência absoluta e relativa, como também a apresentação dos dados por meio de gráficos. Já para os enfermeiros, foi realizada a descrição detalhada da atividade consulta, considerando o volume de observações deste profissional.

### Aspectos éticos

O estudo foi aprovado pelo comitê de ética da instituição responsável (CAAE: 23388819.9.0000.0071), em 28 de novembro de 2019, e pela instituição de saúde do município estudado (CAAE: 23388819.9.3001.0086), em 05 de janeiro de 2020.

## Resultados

Um total de 82 (87,00%) profissionais realizaram pelo menos uma atividade de telessaúde, sendo eles: 10 cirurgiões-dentistas, cinco técnicos de saúde bucal, um auxiliar de saúde bucal, nove médicos, 21 enfermeiros, 12 auxiliares de enfermagem, 16 ACS e oito profissionais da eMulti (um nutricionista, um assistente social, um farmacêutico, um agente de promoção ambiental, dois psicólogos, um fonoaudiólogo e um fisioterapeuta).

Durante o período de acompanhamento da jornada de trabalho destes profissionais, foram observadas 632 atividades de telessaúde, que corresponderam a quase 3,00% do total das atividades realizadas. Em relação ao volume total de atividades de telessaúde dentre todas as atividades observadas, a equipe eMulti foi a que teve a maior parte de suas atividades realizadas no formato de telessaúde (7,53%), seguida dos enfermeiros (3,93%). Em relação às modalidades de telessaúde, 60,28% das atividades foram conduzidas por meio de ligações de voz, 32,28% por aplicativo e 7,44% por meio de videochamadas (Tabela 1).

**Tabela 1 -:** Distribuição das atividades observadas, de acordo com o formato presencial ou telessaúde, realizadas pelos profissionais na APS. São Paulo, SP, Brasil, 2021-2022

**Categorias profissionais**	**Presenciais**		**Telessaúde**							
			**Total**		**Voz**		**Vídeo**		Aplicativo*	
	n	%	n	%	n	%	n	%	n	%
Auxiliar de Enfermagem	4506	99,10	41	0,90	18	43,90	11	26,83	12	29,27
Enfermeiro	6276	96,07	247	3,93	161	65,18	17	6,89	69	27,93
Agente Comunitário de Saúde	4245	96,94	130	3,06	69	53,08	7	5,38	54	41,54
Médico	2810	96,91	87	3,09	70	80,45	2	2,30	15	17,25
Cirurgião-Dentista	1829	98,26	32	1,74	15	46,88	7	21,87	10	31,25
Auxiliar de Saúde Bucal	597	99,00	6	1,00	-	-	-	-	6	100
Técnico de Saúde Bucal	789	97,98	16	2,02	16	100	-	-	-	-
eMulti	969	92,47	73	7,53	32	43,83	3	4,11	38	52,06
**Total**	**22021**	**97,14**	**632**	**2,86**	**381**	**60,28**	**47**	**7,44**	**204**	**32,28**

* Aplicativo = *WhatsApp Business* (mensagem de texto e voz)

Na Figura 1, é possível observar a distribuição das atividades de telessaúde conduzidas pelos profissionais participantes do estudo, classificadas conforme a modalidade de atendimento (voz, vídeo e aplicativo). Notavelmente, seis atividades foram exclusivamente realizadas por meio da modalidade de voz, enquanto oito ocorreram exclusivamente por vídeo. Em relação às visitas domiciliares realizadas remotamente, 67,00% ocorreram na modalidade de voz, enquanto 33,00% utilizaram o aplicativo de comunicação *(WhatsApp Business)*. No contexto das consultas, 91,00% foram conduzidas por voz, 2,00% por vídeo e 7,00% por meio de aplicativo.

No total, foram identificadas 24 categorias de atividades de telessaúde integradas à rotina dos profissionais, divididas em seis de cuidado direto e dezoito de cuidado indireto. De modo abrangente, as atividades remotas mais frequentemente realizadas foram a troca de informações (n=155) e as consultas (n=153). Quanto aos parâmetros de tempo, registram-se 4.669 minutos dedicados às atividades de telessaúde, distribuídos entre 1.355 minutos de cuidado direto, 2.988 minutos de cuidado indireto, 31 minutos de espera e 295 minutos de atividades associadas (Tabela 2).

**Figura 1 -:** Porcentagem do tempo total das atividades de telessaúde de acordo com a modalidade utilizada para o atendimento (ligação de voz, ligação de vídeo e aplicativo de mensagens). São Paulo, SP, Brasil, 2021-2022

*Aplicativo = *WhatsApp Business*(mensagem de texto e voz)

A consulta destacou-se como a atividade de telessaúde de maior tempo dedicado pelos profissionais, totalizando 863 minutos observados entre todos os profissionais, sendo mais frequentemente conduzida por médicos (46,47%) e, em seguida, por enfermeiros (43,92%). O tempo médio despendido pelos enfermeiros para realizar uma consulta de modo remoto foi de 4,86 minutos e de 6,17 minutos para consulta médica.

As atividades de telessaúde relacionadas à Vigilância em Saúde totalizaram 552 minutos, dos quais 62,14% foram conduzidos por enfermeiros. Além disso, dentre as atividades de telessaúde realizadas pelos participantes (n=632), os profissionais da equipe de enfermagem (auxiliares de enfermagem e enfermeiros) se destacaram, totalizando 288 ações, o que representa aproximadamente 46,00% do total de atividades de telessaúde observadas. A atividade de troca de informações sobre cuidados de saúde e/ou serviço de saúde teve 446 minutos de realização em telessaúde, nos quais os enfermeiros e a eMulti foram os que mais realizaram este tipo de atividade, sendo a maioria por meio de ligação de voz (Tabela 2).

**Tabela 2 -:** Frequência, tempo total em minutos, tempo médio em minutos e porcentagem de tempo das atividades de telessaúde realizadas pelos profissionais. São Paulo, SP, Brasil, 2021-2022

Atividades	N Total	TT*	Auxiliar de Enfermagem				Enfermeiro			Agente Comunitário de Saúde			Médico			Cirurgião-Dentista			eMulti	
			n	MT†	% do TT*	n	TM†	% do TT*	n	MT†	% do TT*	n	MT†	% do TT*	n	MT†	% do TT*	n	MT†	% do TT*
Assistência em exames/procedimentos	1	4.00	1	4.00‡	100	-	-	-	-	-	-	-	-	-	-	-	-	-	-	-
Atendimento à Demanda Espontânea	6	26.00	1	4.00‡	15.38	-	-	-	-	-	-	1	6.00‡	23.07	-	-	-	4	4.00	61.53
Consulta	153	863.00	-	-	-	78	4.86	43.92	-	-	-	65	6.17	46.47	4	8.00	3.70	6	8.50	5.91
Controle de Imunização/Vacinação	5	40.00	2	16.50	82.50	3	2.33	17.50	-	-	-	-	-	-	-	-	-	-	-	-
Orientação quanto ao Sistema de Saúde	79	290.00	3.80	6.55	30	3.80	39.31	41	3.59	50.69	-	-	-	-	-	-	-	1	3.00‡	1.03
Visita Domiciliar	43	132.00	-	-	-	-	-	-	43	3.07	100	-	-	-	-	-	-	-	-	-
Ações educativas dos trabalhadores de saúde	6	501.00	-	-	-	2	65.50	26.14	1	69.00‡	13.77	-	-	-	2	103.00	41.11	1	95.00‡	18.96
Apoio ao Estudante	2	8.00	-	-	-	2	4.00	100	-	-	-	-	-	-	-	-	-	-	-	-
Avaliação de desempenho	1	4.00	1	4.00‡	100	-	-	-	-	-	-	-	-	-	-	-	-	-	-	-
Controle de Suprimentos	3	15.00	3	5.00	100	-	-	-	-	-	-	-	-	-	-	-	-	-	-	-
Desenvolvimento de processos e protocolos de cuidado	2	153.00	-	-	-	1	63.00‡	41.18	-	-	-	1	6.00‡	23.07	-	1	90.00‡	58.82	-	-
Desenvolvimento da saúde comunitária	1	10.00	-	-	-	-	-	-	-	-	-	-	-	-	-	-	-	1	10.00‡	100
Documentação	11	172.00	1	9.00‡	5.23	1	5.00‡	2.90	5	23.80	69.18	2	8.50	9.88	-	-	-	2	11.00	12.79
Identificação de Risco	2	126.00	2	63.00	100	-	-	-	-	-	-	-	-	-	-	-	-	-	-	-
Organização do Processo de Trabalho	84	351.00	4	3.25	3.70	30	3.37	28.77	14	3.93	15.67	10	3.30	9.40	11	5.00	15.67	9	2.67	6.83
Pesquisa Científica	3	184.00	-	-	-	-	-	-	-	-	-	-	-	-	3	61.33	100	-	-	-
Referência e Contrarreferência	2	22.00	-	-	-	-	-	-	-	-	-	-	-	-	1	11.00‡	50	1	11.00‡	50
Reunião administrativa	5	257.00	-	-	-	3	45.33	52.92		-	-	-	-	-	1	61.00‡	23.74	1	60.00‡	23.34
Reunião para avaliação dos cuidados multiprofissionais	5	184.00	-	-	-	3	21.33	34.78	1	64.00‡	34.78	-	-	-	-	-	-	1	56.00‡	30.44
Supervisão Segurança	1	3.00	1	3.00‡	100	-	-	-	-	-	-	-	-	-	-	-	-	-	-	-
Troca de Informações sobre cuidados de saúde e/ou serviço de saúde	155	446.00	12	2.17	5.83	72	2.18	35.20	13	3.46	10.09	4	1.00	0.89	4	5.24	4.70	43	4.00	38.56
Vigilância em Saúde	25	552.00	4	34.50	25.00	14	24.50	62.14	1	14‡	2.54	1	15.00‡	2.72	3	9.66	1.63	2	6.50	2.35
Tempo de Espera	7	31.00	-	-	-	2	4.00	25.80	1	11‡	35.48	3	1.67	16.13	-	-	-	1	7.00‡	22.58
Atividade Associada	30	295.00	4	2.75	3.73	6	5.33	10.85	10	3.70	12.54	1	2.00‡	0.68	2	3.50	2.37	-	-	-

*Tempo total em minutos; †Tempo médio em minutos; ‡Quando a atividade foi realizada apenas uma vez, foi utilizado o tempo total

Na Tabela 3, são descritas em detalhes as atividades de consulta conduzidas pelos enfermeiros, categorizadas de acordo com a modalidade. Entre os tipos de consultas, aquelas de acompanhamento e relacionadas à hipertensão/diabetes se destacaram, representando 22,70% e 17,73%, respectivamente. Quanto ao local de realização, o consultório foi o mais frequente, abrangendo 66,23% das consultas, enquanto a ação mais observada durante as consultas foi a anotação, correspondendo a 44,51%, seguida pelo agendamento, que representou 15,09%. Nota-se que, de uma maneira geral, a maioria das consultas realizadas de modo remoto foi conduzida na modalidade de voz.

**Tabela 3 -:** Frequência e porcentagem das atividades de consulta na modalidade de telessaúde realizada pelos enfermeiros. São Paulo, SP, Brasil, 2021-2022

	**n (%)**	**Modalidade**		
		**Voz**	**Vídeo**	Aplicativo*
		n (%)	n (%)	n (%)
**Tipo de consulta** ^†^				
Primeira consulta	7 (4,96)	7 (100,00)	-	-
Acompanhamento	32 (22,70)	27 (84,38)	1 (3,12)	4 (12,50)
Puericultura	1 (0,71)	1 (100,00)	-	-
Saúde da mulher	5 (3,55)	5 (100,00)	-	-
Hipertensão arterial e Diabetes Melittus	25 (17,73)	25 (100,00)	-	-
Saúde mental	1 (0,71)	1 (100,00)	-	-
Entrega de exames	15 (10,64)	14 (93,33)	-	1 (6,70)
Outros	55 (39,01)	55 (100,00)	-	-
**Local da consulta**				
Consultório	51 (66,23)	39 (76,47)	1 (1,96)	11 (21,57)
Sala de procedimento	4 (5,19)	4 (100,00)	-	-
Sala de regulação	1 (1,30)	1 (100,00)	-	-
Outros	21 (27,27)	21 (100,00)	-	-
**Ações realizadas dentro da consulta** ^†^				
Anamnese	23 (21,7)	22 (95,65)	1 (4,35)	-
Exame físico	1 (0,94)	-	1 (100)	-
Prescrição medicamentosa	3 (2,83)	2 (66,67)	-	1 (3,33)
Solicitação de exames	3 (2,83)	2 (66,67)	1 (3,33)	-
Matriciamento com eMulti ^‡^	1 (0,94)	1 (100,00)	-	-
Encaminhamento para especialista	4 (3,77)	4 (100,00)	-	-
Encaminhamento para eMulti ^‡^	2 (1,89)	2 (100,00)	-	-
Agendamento de consulta	16 (15,09)	14 (87,5)	-	2 (12,50)
Anotação	44 (41,51)	43 (97,73)	1 (2,27)	-
Troca de receita	8 (7,55)	7 (87,50)	-	1 (12,50)
Atividade de prevenção	1 (0,94)	1 (100,00)	-	-

*Aplicativo = *WhatsApp Business*(mensagem de texto e voz); ^†^Variáveis com mais de uma opção de resposta; ^‡^Equipe multidisciplinar

## Discussão

Este estudo forneceu um retrato da prática de telessaúde na APS em um município brasileiro. As categorias profissionais que tiveram maior parte de suas atividades realizadas no formato de telessaúde foram as equipes eMulti e de enfermagem. Todavia, a maior contribuição em termos de volume de telessaúde foi realizada pela equipe de enfermagem.

Em relação ao tempo total dedicado ao trabalho em telessaúde, embora os profissionais realizem diversas atividades remotas relacionadas aos aspectos gerenciais, educativos, de pesquisa e assistencial, as consultas representaram a maior parcela de tempo, bem como alta frequência, seguidas por ações de vigilância em saúde, educação permanente e troca de informações entre os profissionais. Ainda, destaca-se que a modalidade mais comum de telessaúde na APS foi por meio de ligações de voz.

As evidências vêm demostrando que as consultas realizadas por telefone e videoconferência são tão eficazes quanto as presenciais para melhorar os resultados clínicos em adultos que frequentam serviços de APS^(2)^. Todavia, apesar da transição das consultas presenciais para telessaúde ter acontecido de modo intenso durante a pandemia, existem preocupações a respeito da sua utilização, pois há estudo que sugere potenciais disparidades raciais, geográficas e outras no acesso aos cuidados^(20)^.

A telessaúde é uma ferramenta potente para ampliação do acesso às consultas dos profissionais de APS e, quando incorporada, busca garantir um acompanhamento contínuo e facilitar o acesso das pessoas com dificuldades de locomoção até o serviço de saúde, seja por barreira física ou de organização, como os horários de atendimento. Por conseguinte, a implementação de atividades de telessaúde na APS deve incorporar competências e processos relacionados à equidade na saúde digital^(21)^.

Os resultados mostraram que a consulta de enfermagem está entre as práticas de telessaúde que mais despendem tempo do enfermeiro na APS, todavia ainda são realizadas em sua maioria pela modalidade de voz. Deste modo, um estudo sinalizou a importância do avanço e instrumentalização para incorporação do processo de enfermagem na abordagem remota^(22)^.

Estudo realizado no Canadá mostrou que os enfermeiros consideraram a pandemia como o principal catalisador para a implementação de teleconsultas, contudo, após o pico da pandemia, as consultas presenciais foram gradualmente substituindo as teleconsultas, sugerindo soluções concretas para incentivar a sua implementação após a pandemia, tais como: integração das teleconsultas nos fluxos de trabalho existentes, de modo que elas possam proporcionar uma vantagem sobre as consultas presenciais; adoção de políticas e protocolos para facilitar a integração das modalidades de teleconsulta no cuidado, bem como direcionamento das pessoas usuárias para o profissional mais adequado para satisfazer as necessidades; por fim, a educação e a formação dos enfermeiros de modo contínuo e permanente, adaptadas às necessidades e com tecnologia de utilização mais simples^(23)^.

A proposta de Serviços de Telessaúde como apoio à Educação Permanente vem sendo discutida ao longo dos anos, no Brasil^(24-25)^, apresentando o potencial para ampliação do acesso dos profissionais às ações formativas e ao desenvolvimento. Categorias profissionais como cirurgiões-dentistas e enfermeiros se destacam no volume de tempo, mas a educação permanente via telessaúde ainda é pouco expressiva na rotina de práticas dos auxiliares de enfermagem, demostrando uma oportunidade em potencial para inserção da telessaúde em todas as categorias profissionais, tal qual a necessidade do desenvolvimento desta competência, para que a equipe da APS possa utilizar a telessaúde como uma ferramenta potente para qualificação do processo de trabalho, bem como fortalecimento da coordenação do cuidado, acesso e vínculo entre as pessoas usuárias, famílias e comunidade.

Destaca-se que a troca de informações foi a atividade mais recorrentemente conduzida de forma remota por todos os profissionais, representando uma das práticas mais frequentes na modalidade de telessaúde adotada pela equipe eMulti. Devido à sua natureza de curta duração, ao analisarmos o volume de tempo dedicado, essa atividade posicionou-se entre as quatro principais. Com efeito, as TICs oferecem uma ampla gama de possibilidades para interatividade e troca entre profissionais, caracterizando-se como uma importante ferramenta de suporte para tomadas de decisões compartilhada^(26)^.

Ações oportunizadas pelas tecnologias de cuidado remoto, como a vigilância em saúde, foram incorporadas nas atividades de todos os profissionais observados, mas destacadamente os auxiliares de enfermagem e enfermeiro foram quem despenderam mais tempo de trabalho. Nesse sentido, enfermeiros canadenses relatam que antes da pandemia as ações de telessaúde realizadas por eles na APS estavam mais direcionadas para acompanhamento de resultados de exames e de algumas doenças crônicas por telefone^(23)^, características também encontradas na realidade brasileira. Todavia, elas se intensificaram com o período pandêmico, como o monitoramento dos casos e resultados de exame, além da rápida adequação realizada pelas equipes com a incorporação das TICs^(27)^. Pesquisa realizada no estado da Bahia demostrou a importância da telessaúde e das tecnologias digitais de saúde como centrais para o monitoramento da propagação de outras doenças emergentes e reemergentes^(28)^.

Além disso, embora a ligação entre o clima e a saúde venha sendo pesquisada nos últimos anos e tenha demostrado os impactos das mudanças climáticas na saúde^(29)^, poucas investigações discutem como essas mudanças climáticas em curso podem impactar as atividades e a atuação de profissionais de saúde na APS, como os ACS. Assim, atividades de telessaúde, para este profissional, poderão ser ferramentas estratégicas para viabilizar visitas domiciliares frente aos fenômenos como ondas de calor, ondas de frio, inundações, secas e outros eventos climáticos extremos que podem impactar a saúde direta e indiretamente.

Neste estudo, observou-se que a modalidade de voz foi a mais frequentemente utilizada durante as atividades de telessaúde. Estudo prévio que comparou dois métodos de telessaúde na APS (voz e vídeo) indicou que, em termos de duração, conteúdo e qualidade da consulta, ambos são semelhantes^(30-31)^. No entanto, a videoconferência pode oferecer uma vantagem, pois permite ao profissional de saúde visualizar as pessoas, contribuindo para o estabelecimento de uma relação mais sólida e aprimorando a comunicação entre ambas as partes^(9)^. Porém, é mais comum ocorrerem problemas técnicos e desigualdades no acesso e no uso de dispositivos digitais com câmeras em comparação com as chamadas apenas por voz^(32)^. Estudo realizado na Austrália apontou que, apesar da preferência de legisladores pela videoconsulta, a maioria das consultas de telessaúde foi realizada por telefone, sugerindo que ainda existem barreiras às videoconsultas^(33)^.

Assim, a adequação dos serviços de telessaúde, considerando ligação de vídeo ou voz é complexa e está ligada a diferentes fatores, como preferências das pessoas, acesso digital, condições de saúde específicas e as necessidades dos membros das equipes. Em estudo conduzido nos Estados Unidos, profissionais de saúde de uma equipe de cuidados primários destacaram a relevância das preferências das pessoas usuárias ao adaptar o teleatendimento, algumas pessoas optaram por encontros virtuais devido à conveniência em termos de custo e tempo de deslocamento, enquanto outros enfrentaram desafios como acesso limitado à internet ou ausência de um aparelho telefônico^(9)^.

Na perspectiva das pessoas usuárias dos serviços de saúde, destacam-se algumas vantagens dos serviços de telessaúde, como: facilidade do acesso aos cuidados de saúde, redução do tempo de espera e a oferta de cuidado de saúde sem risco de exposição à COVID-19, especialmente quando as taxas de transmissão e infecção eram elevadas^(34)^. No entanto, existem desafios a serem superados, tanto para as pessoas usuárias dos serviços de saúde quanto para os profissionais de saúde. Para as pessoas, esses desafios incluem dificuldades de acesso e utilização de tecnologias, bem como preocupações sobre o envolvimento na expectativa das pessoas usuárias e a prestação de atendimento virtual para populações rurais, idosas ou com deficiência cognitiva. Já para os profissionais, envolvem a impossibilidade de realizar exames físicos, a limitação do contato pessoal e do toque, as dificuldades de diagnóstico na ausência de exames físicos, as restrições tecnológicas, a manutenção da relação terapêutica e a garantia de paridade de pagamento entre visitas presenciais e virtuais^(9)^.

Além disso, deve-se considerar que o serviço de telessaúde na APS foi fortalecido com a pandemia de COVID-19, por meio de experiências de readaptação dos serviços oferecidos. Assim, buscou-se um novo caminho para continuidade do cuidado à população, com ações que perpassaram desde readequações na infraestrutura e fluxos de atendimentos até a telemedicina como alternativa de modalidade de atendimento e monitoramento dos casos por meio de aplicativos e via telefone^(35)^. Porém, apesar das experiências fortalecerem oportunidades para a consolidação da telessaúde e o aperfeiçoamento do SUS, após este período, debates apresentavam questionamento sobre a permanência de telessaúde, dado o retorno das atividades presenciais, a demanda crescente nos serviços de APS e as mudanças do processo de trabalho, que não foram acompanhadas de discussões sobre o novo dimensionamento da força de trabalho em APS.

Deste modo, estudo mostrou que pouco se conhece sobre o estado real ou ideal das necessidades da força de trabalho de telessaúde em países de baixo e médio rendimento^(36)^. Além disso, escassos estudos apresentam parâmetros de tempo, como os encontrados por esta pesquisa, que possam subsidiar o planejamento e dimensionamento da força de trabalho em saúde de modo técnico científico.

Sob essa perspectiva, investigação realizada em Gana utilizou a metodologia *Workload Indicators of Staffing Need* para avaliar a suficiência da força de trabalho em saúde digital e estimar a escassez/excesso de pessoal e a pressão da carga de trabalho, pois pouco se sabe sobre a combinação necessária e a dimensão da força de trabalho que seria necessária para gestão eficaz de um sistema de saúde digital em um serviço de saúde, encontrando, então, números inadequados de força de trabalho e uma combinação desequilibrada de competências^(36)^.

Por fim, este estudo apresenta algumas limitações que devem ser consideradas. Primeiramente, a coleta de dados ocorreu durante o período da pandemia de COVID-19, o que pode ter influenciado nas atividades realizadas pela equipe de saúde, refletindo uma adaptação temporária às circunstâncias pandêmicas. Além disso, a metodologia adotada, baseada na observação, pode não ter capturado de forma completa as atividades que foram executadas rapidamente ou que envolviam múltiplas tarefas simultâneas. Tais aspectos podem ter gerado subestimações ou omissões no registro das atividades, limitando a generalização dos resultados.

Apesar das limitações, os resultados deste estudo trazem importantes implicações para a prática dos serviços de saúde. A utilização da observação direta por observadores externos, com técnica *time motion*, proporcionou dados mais precisos e confiáveis em comparação com métodos subjetivos, como o autorrelato sobre as atividades realizadas pelos profissionais de saúde. Ao fornecer um panorama detalhado e inédito do tempo dedicado à telessaúde, em especial da categoria de enfermagem, o estudo contribui para o debate científico sobre a incorporação dessa tecnologia na APS. A telessaúde, uma tecnologia com grande potencial para ampliar acesso às APS, mostra-se como uma ferramenta essencial para a qualificação do trabalho e para o fortalecimento da coordenação do cuidado no SUS.

## Conclusão

Em suma, as atividades de telessaúde realizadas pelos profissionais da APS são diversas, integrando atividades relacionadas às práticas gerenciais, educativas, de pesquisa e assistencial, distribuídas em diferentes proporções na jornada de trabalho. Destacam-se as atividades que concentraram maior tempo da jornada de trabalho, tais como consulta, vigilância em saúde, ações educativas dos trabalhadores de saúde e troca de informações sobre cuidados de saúde e/ou serviço de saúde, em sua maioria realizadas por meio de ligação de voz. Além disso, o estudo apresenta padrões de tempo médio das atividades de telessaúde, subsidiando a discussão do planejamento e dimensionamento da força de trabalho em saúde digital, no contexto da APS.

# Translation: Telesalud en la atención primaria de salud: un estudio de las actividades y el tiempo dedicado por los profesionales*

**DOI:** 10.1590/1518-8345.7255.4499

**Authors:** Debora Bernardo, Daiana Bonfim, Leticia Yamawaka de Almeida, Andrea Liliana Vesga-Varela, Natalia Martins Bonassi, Lorrayne Belotti

**Affiliations:** 1 Hospital Israelita Albert Einstein, Centro de Estudos, Pesquisa e Prática em Atenção Primária à Saúde e Redes, São Paulo, SP, Brasil; 2 Becaria de la Associação Brasileira de Gestão em Projetos do Hospital Israelita Albert Einstein, São Paulo, SP, Brasil; 3 Hospital Israelita Albert Einstein, Saúde Digital, São Paulo, SP, Brasil

**Keywords:** Enfermería, Atención Primaria de Salud, Telemedicina, Estudios de Tiempo y Movimiento, Servicios de Salud, Práctica Profesional

## Abstract

describir las actividades de telesalud realizadas por los profesionales en la Atención Primaria de Salud (APS).

estudio observacional descriptivo, con uso de la metodología *time-motion*. La muestra estuvo compuesta por 31 equipos de Salud de la Familia, 14 equipos de Salud Bucal y dos equipos multidisciplinarios que actúan en la APS. Para la recolección de datos se utilizó un instrumento de carga de trabajo adaptado. Se realizó el análisis descriptivo de los datos considerando el tiempo total, tiempo promedio y porcentaje de las actividades según la categoría profesional y la modalidad de telesalud.

se observaron 632 actividades de telesalud, representando el 2,67% del total de actividades. La modalidad más utilizada fue la llamada de voz (60,28%). El tiempo promedio de la consulta de enfermería fue de 4,86 minutos y de 6,17 minutos para consulta médica. El mayor número de actividades y de tiempo dedicado a telesalud en la APS fue realizado por el equipo de enfermería.

los profesionales de la APS realizan diversas actividades de telesalud, predominantemente por medio de llamadas de voz con una significativa participación del equipo de enfermería. Además, el estudio presenta el tiempo dedicado y la distribución de las actividades, apoyando las discusiones sobre la planificación y dimensionamiento de la fuerza de trabajo en salud digital.

## Destacados

**(1)** Las prácticas de telesalud en la APS se realizan mediante llamadas de voz. **(2)** El equipo de enfermería lleva a cabo el mayor volumen de actividades de telesalud en la APS. **(3)** Las consultas representaron la mayor parte del tiempo y de la frecuencia de las actividades de telesalud en la APS.

## Introducción

Las rápidas transformaciones en las tecnologías de telecomunicación (TICs) han ampliado la posibilidad de su uso, así como su importancia para el acceso a la salud. Estos cambios no solo han influido en los métodos tradicionales de atención y prestación de servicios de salud, sino que también han dado origen a la telesalud, una herramienta que puede mejorar significativamente el acceso a los servicios de salud^(1)^. Este avance puede representar una respuesta ágil a los desafíos contemporáneos, proporcionando un enfoque más eficiente e inclusivo en la oferta de cuidados de salud^(2)^.

En este contexto, la telesalud se configura como una importante estrategia en la oferta remota de servicios de salud, promoviendo la comunicación instantánea entre el individuo y el profesional de salud mediante recursos electrónicos de audio y video. Dicha estrategia tiene como objetivo mitigar los obstáculos geográficos, permitiendo la colaboración entre profesionales de salud y el uso de dispositivos electrónicos para intervención o telediagnóstico^(3-4)^.

Aunque la telesalud no es un concepto nuevo y ha sido utilizada en muchas organizaciones y sistemas de salud, la pandemia de COVID-19 intensificó su uso en diferentes entornos de atención de salud^(5)^. Su incorporación en los cuidados primarios, por ejemplo, cobró relevancia debido a la necesidad de monitorear a las personas con síntomas respiratorios y asegurar la continuidad de los cuidados para una gestión exitosa de enfermedades crónicas^(6)^.

En Brasil, la telesalud, reglamentada por la Ley 14.510 del 27 de diciembre de 2022^(7)^, tiene como objetivo ampliar el acceso de la población al Sistema Único de Salud (SUS), complementando las acciones presenciales. Asimismo, abarca actividades como acompañamiento, diagnóstico, tratamiento y vigilancia epidemiológica, con el fin de proporcionar asistencia, prevención, promoción, educación, investigación y gestión en salud^(8)^.

En el ámbito de la Atención Primaria de Salud (APS), proporciona beneficios como el acceso continuo a servicios de salud en áreas remotas; la promoción de la autogestión y capacitación de las personas; la reducción de costos; y la minimización de la necesidad de desplazamientos para cuidados de salud^(9)^. Estos elementos contribuyen a alcanzar la equidad en el acceso a la salud, especialmente en áreas remotas y rurales. Además, facilita la comunicación entre las personas usuarias, cuidadores familiares y profesionales de salud, promoviendo el intercambio de información crítica^(1)^.

Un estudio realizado en Brasil, que examinó la implementación del Programa Nacional TeleSalud Brasil Redes en la APS, destacó la teleeducación como la modalidad más frecuentemente adoptada por los equipos de salud. Adicionalmente, se constató que las mayores tasas de uso ocurrieron en las regiones Sur y Sudeste del país, en municipios con hasta 30 mil habitantes, con al menos un médico presente y un teléfono disponible en el establecimiento. Aspectos estructurales, como la presencia de internet y salas multifuncionales, no tuvieron un impacto significativo en la adopción de estas tecnologías^(10)^.

Sin embargo, factores como la disponibilidad de un dispositivo habilitado para video, una conexión de internet razonablemente fuerte y alfabetización digital pueden ser considerados barreras para la adhesión de algunas personas a este tipo de atención^(11)^. Además, la comunicación sobre los síntomas puede ser un desafío para las personas usuarias del servicio de salud, requiriendo un acompañamiento continuo para evitar posibles daños causados por condiciones no diagnosticadas^(12)^. Adicionalmente, una investigación que examinó la perspectiva de profesionales y gestores de la APS sobre un programa de telesalud en una capital brasileña identificó desafíos que incluían problemas en el uso del sistema, cuestiones relacionadas con la infraestructura, dificultades prácticas en la implementación del programa, obstáculos en la integración a la cultura organizacional y falta de soporte gerencial^(13)^.

Aunque la literatura ha abordado la incorporación de la telesalud en la APS, principalmente en el contexto de la pandemia de COVID-19, existe una brecha significativa en cuanto a la descripción detallada de las actividades específicas realizadas por los profesionales de salud, cuánto tiempo de trabajo en la APS ocupa la telesalud y los parámetros de tiempo promedio dedicado a cada una de ellas. Un estudio anterior se enfocó en la implementación de programas de telesalud y en los desafíos estructurales y tecnológicos enfrentados, sin avanzar en un análisis detallado de las prácticas de telesalud en el día a día de los equipos de salud, con un enfoque en la planificación y optimización de la fuerza de trabajo en salud digital^(10)^.

Ante este escenario, se hace esencial la identificación de las actividades de telesalud desempeñadas por los profesionales de salud en la APS en el contexto brasileño, situando esta práctica en el ámbito del proceso de trabajo de los equipos y promoviendo la ampliación del debate sobre la planificación de la fuerza de trabajo frente a la introducción de nuevas herramientas en la rutina del cuidado en salud. Así, el presente estudio tiene como objetivo describir las actividades de telesalud realizadas por los profesionales en la Atención Primaria de Salud.

## Método

### Diseño del estudio

Estudio observacional descriptivo, en el cual se utilizó la técnica *time-motion*, una técnica de observación continua que permite la recolección de datos de manera detallada con el objetivo de determinar las actividades y el volumen de tiempo que los profesionales dedican a su realización durante la jornada laboral^(14)^.

### Escenario

El estudio se llevó a cabo en cinco Unidades Básicas de Salud (UBS), ubicadas en la región Sur de un municipio de gran tamaño en el estado de São Paulo (SP), Brasil. Estos servicios, ubicados en territorios vulnerables^(15)^, cuentan con aproximadamente 115 mil personas registradas, operan de lunes a viernes (de 7h a 19h) y disponen de 31 equipos de Salud de la Familia, 14 equipos de Salud Bucal y dos equipos multidisciplinarios (eMulti). Todos los equipos de salud disponen de un teléfono móvil modelo *smartphone* proporcionado por la institución gestora para facilitar la telesalud durante la rutina laboral desde el inicio del período de pandemia de COVID-19.

### Población

La población del estudio estuvo conformada por profesionales de salud con nivel superior y medio de educación, quienes cumplían con los criterios de inclusión y fueron invitados a participar en el estudio. Estos profesionales estaban vinculados a los equipos mencionados y distribuidos en las siguientes categorías: 12 cirujanos dentistas, cinco técnicos de salud bucal, tres auxiliares de salud bucal, 11 médicos, 21 enfermeros, 15 auxiliares de enfermería, 18 agentes comunitarios de salud (ACS) y profesionales de la eMulti (un nutricionista, un trabajador social, un farmacéutico, un agente de promoción ambiental, dos psicólogos, un fonoaudiólogo, un fisioterapeuta y un terapeuta ocupacional).

Inicialmente se realizó el contacto con la coordinación de cada UBS para el reclutamiento de los participantes. Con la anuencia de los coordinadores locales y conforme a los protocolos sanitarios del período, los investigadores llevaron a cabo un encuentro en grupo con los profesionales de salud en cada uno de los servicios para la presentación de la investigación y la formalización de la invitación a los profesionales de salud. En esa ocasión, se contextualizaron los aspectos éticos de la investigación, y en ese momento, se invitó a los profesionales a acceder a un formulario electrónico mediante un *QR code* para la lectura del Consentimiento Informado. También cabe señalar que, para aquellos profesionales que, por alguna razón, no participaron en estos encuentros, se realizó, cuando fue posible, el contacto de forma individual para la presentación de la propuesta y formalización de la invitación a participar en el estudio.

Aquellos que manifestaron interés y conformidad en participar en la investigación fueron dirigidos al llenado de un cuestionario con elementos relacionados con el perfil sociodemográfico y laboral. En una segunda fase, se acordó con la coordinación de los servicios y los profesionales de salud el período de recolección de datos y la definición de un cronograma para cada unidad.

### Instrumentos de recolección de datos y variables

El instrumento de medición de la carga de trabajo^(16)^ fue adaptado y utilizado para orientar el registro de las actividades realizadas por los profesionales de salud. En el presente estudio, se consideró un listado de 41 actividades, organizadas en intervenciones de cuidado directo e indirecto, actividades asociadas y tiempo de espera, tal como se describe a continuación:

Cuidado directo: son actividades realizadas en presencia del usuario/familia/comunidad. Ejemplos: asistencia en exámenes/procedimientos; atención a demanda espontánea; consulta, control de inmunización/vacunación; orientación sobre el sistema de salud y visita domiciliaria^(16)^.

Cuidado indirecto: son actividades realizadas sin la presencia del usuario/familia/comunidad, pero en su beneficio. Ejemplos: acciones educativas de los trabajadores de salud; apoyo al estudiante; evaluación de desempeño; control de suministros; desarrollo de procesos y protocolos de cuidado; desarrollo de la salud comunitaria; documentación; identificación de riesgo; organización del proceso de trabajo; investigación científica; referencia y contrarreferencia; reunión administrativa; reunión para evaluación de los cuidados multiprofesionales; supervisión de seguridad; intercambio de información sobre cuidados de salud y/o servicio de salud; vigilancia en salud^(16)^.

Actividad asociada: son actividades que pueden ser realizadas por otras categorías profesionales, pero que el profesional de salud asume^(16)^. Ejemplos: buscar material; buscar expediente; limpieza de la unidad; buscar a otro profesional en la unidad; buscar sala, entre otros.

Tiempo de espera: cuando el profesional está en su puesto de trabajo disponible para la atención, esperando al usuario y/o a otro profesional^(16)^.

Cabe destacar que, para la identificación de las actividades de telesalud durante la observación, se incluyó un ítem en el instrumento para registrar el formato en que se desarrollaba la actividad (presencial o remota). Para las actividades remotas, se mapearon como modalidades tecnológicas las siguientes opciones: llamada de voz, videollamada o mensajes instantáneos de texto o voz por medio de aplicaciones de comunicación (*WhatsApp Business*).

### Recolección de datos

La recolección de datos se realizó en dos momentos para cumplir con las recomendaciones sanitarias del período de pandemia de COVID-19, en los períodos de marzo a agosto de 2021, así como de marzo a agosto de 2022. Esta etapa fue llevada a cabo por un equipo de 10 profesionales de nivel superior, becarios, compuesto por cinco enfermeros, dos médicos obstetras, dos cirujanos dentistas y un psicólogo, debidamente entrenados (120 horas teórico-prácticas) por el investigador responsable del desarrollo del proyecto, para realizar una observación directa y continua de la rutina laboral de los profesionales de salud, empleando la técnica *time-motion* ^(17)^. Dada la necesidad de un observador designado para cada profesional, un investigador fue asignado a la función de observador para acompañar a un profesional específico a lo largo de todas las actividades realizadas en una semana típica de trabajo.

La observación, sin interacción ni intervención, se realizó durante 179 días. Comenzando con la llegada del profesional de salud a la UBS y finalizando al concluir el turno de trabajo, que variaba entre seis y ocho horas diarias, dependiendo de la categoría profesional investigada. Los datos eran registrados simultáneamente a la observación en un dispositivo electrónico portátil (*tablet*), que fue utilizado exclusivamente para la investigación y almacenados en el *Research Electronic Data Capture* (REDCap)^(18-19)^, plataforma de recolección y gestión de datos.

En el ámbito de este estudio, se incluyeron las actividades realizadas mediante llamadas de voz, videollamadas o aplicaciones de comunicación por parte de los profesionales de salud vinculados a las UBS que integraron las observaciones.

### Tratamiento y análisis de datos

Se realizó un análisis descriptivo de los datos utilizando el *software* Excel, considerando el tiempo total, el tiempo promedio y la proporción de actividades, teniendo en cuenta las diferentes categorías profesionales y las modalidades de atención de telesalud (llamada de voz, videollamada o mensajes instantáneos de texto o voz). En vista de esto, se construyeron tablas de frecuencia absoluta y relativa, así como la presentación de los datos mediante gráficos. Ahora, para los enfermeros se realizó una descripción detallada de la actividad de consulta, considerando el volumen de observaciones de este profesional.

### Aspectos éticos

El estudio fue aprobado por el comité de ética de la institución responsable (CAAE: 23388819.9.0000.0071), el 28 de noviembre de 2019, y por la institución de salud del municipio estudiado (CAAE: 23388819.9.3001.0086), el 5 de enero de 2020.

## Resultados

Un total de 82 (87,00%) profesionales realizaron al menos una actividad de telesalud, siendo ellos: 10 cirujanos dentistas, cinco técnicos de salud bucal, un auxiliar de salud bucal, nueve médicos, 21 enfermeros, 12 auxiliares de enfermería, 16 ACS y ocho profesionales de la eMulti (un nutricionista, un trabajador social, un farmacéutico, un agente de promoción ambiental, dos psicólogos, un fonoaudiólogo y un fisioterapeuta).

Durante el período de seguimiento de la jornada laboral de estos profesionales, se observaron 632 actividades de telesalud, que correspondieron a casi el 3,00% del total de actividades realizadas. En cuanto al volumen total de actividades de telesalud entre todas las actividades observadas, el equipo eMulti fue el que tuvo la mayor parte de sus actividades realizadas en el formato de telesalud (7,53%), seguido por los enfermeros (3,93%). En cuanto a las modalidades de telesalud, el 60,28% de las actividades se realizaron mediante llamadas de voz, el 32,28% a través de aplicaciones y el 7,44% mediante videollamadas (Tabla 1).

**Tabla 1 -:** Distribución de las actividades observadas, según el formato presencial o telesalud, realizadas por los profesionales en la APS. São Paulo, SP, Brasil, 2021-2022

**Categorías profesionales**	**Presenciales**		**Telesalud**							
			**Total**		**Voz**		**Video**		Aplicación*	
	n	%	n	%	n	%	n	%	n	%
Auxiliar de Enfermería	4506	99,10	41	0,90	18	43,90	11	26,83	12	29,27
Enfermero	6276	96,07	247	3,93	161	65,18	17	6,89	69	27,93
Agente Comunitario de Salud	4245	96,94	130	3,06	69	53,08	7	5,38	54	41,54
Médico	2810	96,91	87	3,09	70	80,45	2	2,30	15	17,25
Cirujano Dentista	1829	98,26	32	1,74	15	46,88	7	21,87	10	31,25
Auxiliar de Salud Bucal	597	99,00	6	1,00	-	-	-	-	6	100
Técnico de Salud Bucal	789	97,98	16	2,02	16	100	-	-	-	-
eMulti	969	92,47	73	7,53	32	43,83	3	4,11	38	52,06
**Total**	**22021**	**97,14**	**632**	**2,86**	**381**	**60,28**	**47**	**7,44**	**204**	**32,28**

*Aplicación = *WhatsApp Business* (mensaje de texto y voz)

En la Figura 1, es posible observar la distribución de las actividades de telesalud realizadas por los profesionales participantes del estudio, clasificadas según la modalidad de atención (voz, video y aplicación). Notablemente, seis actividades se realizaron exclusivamente mediante la modalidad de voz, mientras que ocho se realizaron exclusivamente por video. En cuanto a las visitas domiciliarias realizadas de forma remota, el 67,00% se llevaron a cabo en la modalidad de voz, mientras que el 33,00% utilizaron la aplicación de comunicación (*WhatsApp Business*). En el contexto de las consultas, el 91,00% se realizaron por voz, el 2,00% por video y el 7,00% mediante aplicación.

En total, se identificaron 24 categorías de actividades de telesalud integradas en la rutina de los profesionales, divididas en seis de cuidado directo y dieciocho de cuidado indirecto. De manera general, las actividades remotas más frecuentemente realizadas fueron el intercambio de información (n=155) y las consultas (n=153). En cuanto a los parámetros de tiempo, se registraron 4.669 minutos dedicados a las actividades de telesalud, distribuidos entre 1.355 minutos de cuidado directo, 2.988 minutos de cuidado indirecto, 31 minutos de espera y 295 minutos de actividades asociadas (Tabla 2).

**Tabla 2 -:** Frecuencia, tiempo total en minutos, tiempo promedio en minutos y porcentaje de tiempo de las actividades de telesalud realizadas por los profesionales. São Paulo, SP, Brasil, 2021-2022

Actividades	N Total	TT*	Auxiliar de Enfermería				Enfermero			Agente Comunitario de de Salud			Médico			Cirujano-Dentista			eMulti	
			n	MT†	% del TT*	n	TM†	% del TT*	n	MT†	% del TT*	n	MT†	% del TT*	n	MT†	% del TT*	n	MT†	% del TT*
Asistencia en exámenes/procedimientos	1	4.00	1	4.00‡	100	-	-	-	-	-	-	-	-	-	-	-	-	-	-	-
Atención a la Demanda Espontánea	6	26.00	1	4.00‡	15.38	-	-	-	-	-	-	1	6.00‡	23.07	-	-	-	4	4.00	61.53
Consulta	153	863.00	-	-	-	78	4.86	43.92	-	-	-	65	6.17	46.47	4	8.00	3.70	6	8.50	5.91
Control de Inmunización/Vacunación	5	40.00	2	16.50	82.50	3	2.33	17.50	-	-	-	-	-	-	-	-	-	-	-	-
Orientación sobre el Sistema de Salud	79	290.00	3.80	6.55	30	3.80	39.31	41	3.59	50.69	-	-	-	-	-	-	-	1	3.00‡	1.03
Visita Domiciliaria	43	132.00	-	-	-	-	-	-	43	3.07	100	-	-	-	-	-	-	-	-	-
Acciones educativas de los trabajadores de salud	6	501.00	-	-	-	2	65.50	26.14	1	69.00‡	13.77	-	-	-	2	103.00	41.11	1	95.00‡	18.96
Apoyo al Estudiante	2	8.00	-	-	-	2	4.00	100	-	-	-	-	-	-	-	-	-	-	-	-
Evaluación de desempeño	1	4.00	1	4.00‡	100	-	-	-	-	-	-	-	-	-	-	-	-	-	-	-
Control de Suministros	3	15.00	3	5.00	100	-	-	-	-	-	-	-	-	-	-	-	-	-	-	-
Desarrollo de procesos y protocolos de atención	2	153.00	-	-	-	1	63.00‡	41.18	-	-	-	1	6.00‡	23.07	-	1	90.00‡	58.82	-	-
Desarrollo de la salud comunitaria	1	10.00	-	-	-	-	-	-	-	-	-	-	-	-	-	-	-	1	10.00‡	100
Documentación	11	172.00	1	9.00‡	5.23	1	5.00‡	2.90	5	23.80	69.18	2	8.50	9.88	-	-	-	2	11.00	12.79
Investigación Científica	2	126.00	2	63.00	100	-	-	-	-	-	-	-	-	-	-	-	-	-	-	-
Organización del Proceso de Trabajo	84	351.00	4	3.25	3.70	30	3.37	28.77	14	3.93	15.67	10	3.30	9.40	11	5.00	15.67	9	2.67	6.83
Investigación Científica	3	184.00	-	-	-	-	-	-	-	-	-	-	-	-	3	61.33	100	-	-	-
Referencia y Contrarreferencia	2	22.00	-	-	-	-	-	-	-	-	-	-	-	-	1	11.00‡	50	1	11.00‡	50
Reunión administrativa	5	257.00	-	-	-	3	45.33	52.92		-	-	-	-	-	1	61.00‡	23.74	1	60.00‡	23.34
Reunión para evaluación de cuidados multiprofesionales	5	184.00	-	-	-	3	21.33	34.78	1	64.00‡	34.78	-	-	-	-	-	-	1	56.00‡	30.44
Supervisión de Seguridad	1	3.00	1	3.00‡	100	-	-	-	-	-	-	-	-	-	-	-	-	-	-	-
Intercambio de Información sobre cuidados desalud y/o servicio de salud y/o servicio de salud	155	446.00	12	2.17	5.83	72	2.18	35.20	13	3.46	10.09	4	1.00	0.89	4	5.24	4.70	43	4.00	38.56
Vigilancia en Salud	25	552.00	4	34.50	25.00	14	24.50	62.14	1	14‡	2.54	1	15.00‡	2.72	3	9.66	1.63	2	6.50	2.35
Tiempo de Espera	7	31.00	-	-	-	2	4.00	25.80	1	11‡	35.48	3	1.67	16.13	-	-	-	1	7.00‡	22.58
Actividad Asociada	30	295.00	4	2.75	3.73	6	5.33	10.85	10	3.70	12.54	1	2.00‡	0.68	2	3.50	2.37	-	-	-

*Aplicación = *WhatsApp Business* (mensaje de texto y voz)

**Figura 1 -:** Porcentaje del tiempo total de las actividades de telesalud según la modalidad utilizada para la atención (llamada de voz, videollamada y aplicación de mensajería). São Paulo, SP, Brasil, 2021-2022

*Aplicación = WhatsApp Business (mensaje de texto y voz)

La consulta destacó como la actividad de telesalud con mayor tiempo dedicado por los profesionales, totalizando 863 minutos observados entre todos los profesionales, siendo realizada más frecuentemente por médicos (46,47%) y, en segundo lugar, por enfermeros (43,92%). El tiempo promedio empleado por los enfermeros para realizar una consulta de forma remota fue de 4,86 minutos y de 6,17 minutos para consulta médica.

Las actividades de telesalud relacionadas con la Vigilancia en Salud totalizaron 552 minutos, de los cuales el 62,14% fueron llevados a cabo por enfermeros. Además, entre las actividades de telesalud realizadas por los participantes (n=632), los profesionales del equipo de enfermería (auxiliares de enfermería y enfermeros) se destacaron, totalizando 288 acciones, lo que representa aproximadamente el 46,00% del total de actividades de telesalud observadas. La actividad de intercambio de información sobre cuidados de salud y/o servicio de salud tuvo 446 minutos de ejecución en telesalud, donde los enfermeros y la eMulti fueron quienes realizaron este tipo de actividad con mayor frecuencia, siendo la mayoría mediante llamadas de voz (Tabla 3).

En la Tabla 3, se describen en detalle las actividades de consulta realizadas por los enfermeros, categorizadas según la modalidad. Entre los tipos de consultas, las de seguimiento y las relacionadas con hipertensión/diabetes se destacaron, representando el 22,70% y el 17,73%, respectivamente. En cuanto al lugar de realización, el consultorio fue el más frecuente, abarcando el 66,23% de las consultas, mientras que la acción más observada durante las consultas fue la anotación, correspondiendo al 44,51%, seguida por la programación de citas, que representó el 15,09%. Se observa que, en términos generales, la mayoría de las consultas realizadas de forma remota se llevaron a cabo mediante la modalidad de voz.

**Tabla 3 -:** Frecuencia y porcentaje de las actividades de consulta en la modalidad de telesalud realizadas por los enfermeros. São Paulo, SP, Brasil, 2021-2022

	**n (%)**	**Modalidad**		
		**Voz**	**Video**	Aplicación*
		n (%)	n (%)	n (%)
**Tipo de consulta** ^†^				
Primera consulta	7 (4,96)	7 (100,00)	-	-
Seguimiento	32 (22,70)	27 (84,38)	1 (3,12)	4 (12,50)
Puericultura	1 (0,71)	1 (100,00)	-	-
Salud de la mujer	5 (3,55)	5 (100,00)	-	-
Hipertensión arterial y Diabetes Mellitus	25 (17,73)	25 (100,00)	-	-
Salud mental	1 (0,71)	1 (100,00)	-	-
Entrega de exámenes	15 (10,64)	14 (93,33)	-	1 (6,70)
Otros	55 (39,01)	55 (100,00)	-	-
**Lugar de la consulta**				
Consultorio	51 (66,23)	39 (76,47)	1 (1,96)	11 (21,57)
Sala de procedimientos	4 (5,19)	4 (100,00)	-	-
Sala de regulación	1 (1,30)	1 (100,00)	-	-
Otros	21 (27,27)	21 (100,00)	-	-
**Acciones realizadas dentro de la consulta** ^†^				
Anamnesis	23 (21,7)	22 (95,65)	1 (4,35)	-
Examen físico	1 (0,94)	-	1 (100)	-
Prescripción de medicamentos	3 (2,83)	2 (66,67)	-	1 (3,33)
Solicitud de exámenes	3 (2,83)	2 (66,67)	1 (3,33)	-
Matrización con eMulti ^‡^	1 (0,94)	1 (100,00)	-	-
Derivación a especialista	4 (3,77)	4 (100,00)	-	-
Derivación a eMulti ^‡^	2 (1,89)	2 (100,00)	-	-
Agendamiento de consulta	16 (15,09)	14 (87,5)	-	2 (12,50)
Anotación	44 (41,51)	43 (97,73)	1 (2,27)	-
Cambio de receta	8 (7,55)	7 (87,50)	-	1 (12,50)
Actividad de prevención	1 (0,94)	1 (100,00)	-	-

*Aplicación = *WhatsApp business* (mensaje de texto y voz); ^†^Variables con más de una opción de respuesta; ^‡^Equipo multidisciplinario

## Discusión

Este estudio proporcionó un retrato de la práctica de telesalud en la APS en un municipio brasileño. Las categorías profesionales que realizaron la mayor parte de sus actividades en el formato de telesalud fueron los equipos eMulti y de enfermería. Sin embargo, la mayor contribución en términos de volumen de telesalud fue realizada por el equipo de enfermería.

En cuanto al tiempo total dedicado al trabajo en telesalud, aunque los profesionales realizan diversas actividades remotas relacionadas con los aspectos de gestión, educativos, de investigación y asistenciales, las consultas representaron la mayor parte del tiempo, así como una alta frecuencia, seguidas por acciones de vigilancia en salud, educación continua e intercambio de información entre los profesionales. Todavía, cabe destacar que la modalidad de telesalud más común en la APS fue mediante llamadas de voz.

Las evidencias han demostrado que las consultas realizadas por teléfono y videoconferencia son tan eficaces como las presenciales para mejorar los resultados clínicos en adultos que frecuentan servicios de APS^(2)^. Sin embargo, a pesar de que la transición de las consultas presenciales a telesalud ocurrió de manera intensa durante la pandemia, existen preocupaciones sobre su utilización, ya que un estudio sugiere posibles disparidades raciales, geográficas y otras en el acceso a la atención^(20)^.

La telesalud es una herramienta potente para ampliar el acceso a las consultas de los profesionales de APS y, cuando es incorporada, busca garantizar un seguimiento continuo y facilitar el acceso de las personas con dificultades de movilidad al servicio de salud, ya sea por barreras físicas o de organización, como los horarios de atención. Por tanto, la implementación de actividades de telesalud en la APS debe incorporar competencias y procesos relacionados con la equidad en la salud digital^(21)^.

Los resultados mostraron que la consulta de enfermería está entre las prácticas de telesalud que más tiempo requiere del enfermero en la APS, aunque aún se realizan en su mayoría mediante llamadas de voz. De este modo, un estudio señaló la importancia de avanzar y de instrumentalizar el proceso de enfermería en el abordaje remoto^(22)^.

Un estudio realizado en Canadá mostró que los enfermeros consideraron la pandemia como el principal catalizador para la implementación de teleconsultas, pero que, después del pico de la pandemia, las consultas presenciales gradualmente fueron sustituyendo las teleconsultas, sugiriendo soluciones concretas para incentivar su implementación después de la pandemia, tales como: integración de las teleconsultas en los flujos de trabajo existentes, de modo que puedan proporcionar una ventaja sobre las consultas presenciales; adopción de políticas y protocolos para facilitar la integración de las modalidades de teleconsulta en la atención, así como la derivación de los usuarios al profesional más adecuado para satisfacer sus necesidades; finalmente, la educación y formación continua y permanente de los enfermeros, adaptadas a las necesidades y con tecnología más sencilla de utilizar^(23)^.

La propuesta de Servicios de Telesalud como apoyo a la Educación Continua ha sido discutida a lo largo de los años en Brasil^(24-25)^, mostrando su potencial para ampliar el acceso de los profesionales a las acciones formativas y de desarrollo. Categorías profesionales como cirujanos dentistas y enfermeros destacan en el volumen de tiempo, pero la educación continua por medio de telesalud aún es poco significativa en la rutina de prácticas de los auxiliares de enfermería, mostrando una oportunidad potencial para insertar la telesalud en todas las categorías profesionales, al igual que la necesidad de desarrollar esta competencia, para que el equipo de APS pueda utilizar la telesalud como una herramienta potente para la calificación del proceso de trabajo, así como para fortalecer la coordinación de la atención, el acceso y el vínculo entre los usuarios, familias y comunidad.

Cabe señalar que el intercambio de información fue la actividad más recurrente realizada de forma remota por todos los profesionales, representando una de las prácticas más frecuentes en la modalidad de telesalud adoptada por el equipo eMulti. Debido a su naturaleza de corta duración, al analizar el volumen de tiempo dedicado, esta actividad se ubicó entre las cuatro principales. En efecto, las TIC ofrecen una amplia gama de posibilidades para la interactividad y el intercambio entre profesionales, caracterizándose como una herramienta importante de soporte para la toma de decisiones compartidas^(26)^.

Las acciones facilitadas por las tecnologías de atención remota, como la vigilancia en salud, se incorporaron en las actividades de todos los profesionales observados, aunque fueron los auxiliares de enfermería y los enfermeros quienes dedicaron más tiempo de trabajo. En ese sentido, enfermeros canadienses relatan que antes de la pandemia, las acciones de telesalud realizadas por ellos en la APS estaban más enfocadas en el seguimiento de resultados de exámenes y algunas enfermedades crónicas por teléfono^(23)^, características también observadas en la realidad brasileña. No obstante, se intensificaron durante el período pandémico, como el monitoreo de casos y resultados de exámenes, además de la rápida adaptación realizada por los equipos con la incorporación de las TIC^(27)^. Una investigación realizada en el estado de Bahia demostró la importancia de la telesalud y de las tecnologías digitales de salud como herramientas clave para el monitoreo de la propagación de otras enfermedades emergentes y reemergentes^(28)^.

Además, aunque el vínculo entre el clima y la salud ha sido investigado en los últimos años, y se han demostrado los impactos de los cambios climáticos en la salud^(29)^, pocas investigaciones discuten cómo estos cambios climáticos en curso pueden afectar las actividades y el desempeño de los profesionales de salud en la APS, como los ACS. Así, las actividades de telesalud, para este profesional, pueden ser herramientas estratégicas para viabilizar las visitas domiciliarias frente a fenómenos como olas de calor, olas de frío, inundaciones, sequías y otros eventos climáticos extremos que pueden afectar la salud directa e indirectamente.

En este estudio, se observó que la modalidad de voz fue la más utilizada durante las actividades de telesalud. Un estudio previo que comparó dos métodos de telesalud en la APS (voz y video) indicó que, en términos de duración, contenido y calidad de la consulta, ambos son semejantes^(30-31)^. Sin embargo, la videoconferencia puede ofrecer una ventaja, ya que permite al profesional de salud ver a los usuarios, contribuyendo a establecer una relación más sólida y mejorando la comunicación entre ambas partes^(9)^. Sin embargo, es más común que ocurran problemas técnicos y desigualdades en el acceso y uso de dispositivos digitales con cámaras en comparación con las llamadas solo de voz^(32)^. Además, un estudio realizado en Australia demostró que, aunque los legisladores prefieren las videoconsultas, la mayoría de las consultas de telesalud se realizaron por teléfono, lo que sugiere que aún existen barreras para las videoconsultas^(33)^.

Así, la adecuación de los servicios de telesalud, considerando la llamada de video o voz, es compleja y está vinculada a diferentes factores, como las preferencias de las personas, el acceso digital, las condiciones de salud específicas y las necesidades de los miembros de los equipos. En un estudio realizado en Estados Unidos, los profesionales de salud de un equipo de cuidados primarios destacaron la relevancia de las preferencias de los usuarios al adaptar la teleatención. Algunas personas optaron por encuentros virtuales debido a la conveniencia en términos de costo y tiempo de desplazamiento, mientras que otras enfrentaron desafíos como acceso limitado a internet o la falta de un dispositivo telefónico^(9)^.

Desde la perspectiva de las personas usuarias de los servicios de salud, se destacan algunas ventajas de los servicios de telesalud, como: la facilidad de acceso a los cuidados de salud, la reducción del tiempo de espera y la oferta de atención sin riesgo de exposición a la COVID-19, especialmente cuando las tasas de transmisión e infección eran elevadas^(34)^. Sin embargo, existen desafíos a superar, tanto para las personas usuarias de los servicios de salud como para los profesionales de salud. Para las personas, estos desafíos incluyen dificultades en el acceso y uso de las tecnologías, así como preocupaciones sobre el involucramiento en las expectativas de las personas usuarias y la prestación de atención virtual para poblaciones rurales, personas mayores o con discapacidad cognitiva. Para los profesionales, los desafíos incluyen la imposibilidad de realizar exámenes físicos, la limitación del contacto personal y el tacto, dificultades de diagnóstico en ausencia de exámenes físicos, restricciones tecnológicas, mantenimiento de la relación terapéutica y garantía de paridad de pago entre visitas presenciales y virtuales^(9)^.

Además, se debe considerar que el servicio de telesalud en la APS se fortaleció con la pandemia de COVID-19, mediante experiencias de readaptación de los servicios ofrecidos. Así, se buscó un nuevo camino para la continuidad del cuidado a la población, con acciones que abarcaron desde la readecuación de la infraestructura y los flujos de atención hasta la telemedicina como alternativa de modalidad de atención y monitoreo de casos por medio de aplicaciones y por teléfono^(35)^. Sin embargo, a pesar de que estas experiencias fortalecieron las oportunidades para la consolidación de la telesalud y el perfeccionamiento del SUS, después de este período, surgieron debates sobre la permanencia de la telesalud, dado el regreso de las actividades presenciales, la creciente demanda en los servicios de APS y los cambios en el proceso de trabajo, que no estuvieron acompañados de discusiones sobre la nueva dimensionamiento de la fuerza laboral en APS.

Así, un estudio mostró que se conoce poco sobre el estado real o ideal de las necesidades de la fuerza laboral de telesalud en países de ingresos bajos y medios^(36)^. Además, pocos estudios presentan parámetros de tiempo, como los encontrados por esta investigación, que puedan sustentar el planeamiento y dimensionamiento de la fuerza laboral en salud de forma técnica y científica.

Desde esta perspectiva, una investigación realizada en Ghana utilizó la metodología *Workload Indicators of Staffing Need* para evaluar la suficiencia de la fuerza laboral en salud digital y estimar la escasez/exceso de personal y la presión de la carga de trabajo, ya que se sabe poco sobre la combinación necesaria y la dimensión de la fuerza laboral que sería necesaria para la gestión eficaz de un sistema de salud digital en un servicio de salud, encontrando, entonces, números inadecuados de fuerza laboral y una combinación desequilibrada de competencias^(36)^.

Finalmente, este estudio presenta algunas limitaciones que deben ser consideradas. En primer lugar, la recopilación de datos se realizó durante el período de la pandemia de COVID-19, lo cual puede haber influido en las actividades realizadas por el equipo de salud, reflejando una adaptación temporal a las circunstancias pandémicas. Además, la metodología adoptada, basada en la observación, puede no haber captado de forma completa las actividades que fueron ejecutadas rápidamente o que involucraban múltiples tareas simultáneas. Tales aspectos pueden haber generado subestimaciones u omisiones en el registro de las actividades, limitando la generalización de los resultados.

A pesar de las limitaciones, los resultados de este estudio tienen importantes implicaciones para la práctica de los servicios de salud. La utilización de la observación directa por observadores externos, con la técnica de *time-motion*, proporcionó datos más precisos y confiables en comparación con métodos subjetivos, como el autoinforme, sobre las actividades realizadas por los profesionales de salud. Al proporcionar un panorama detallado e inédito del tiempo dedicado a la telesalud, especialmente en la categoría de enfermería, el estudio contribuye al debate científico sobre la incorporación de esta tecnología en la APS. La telesalud, una tecnología con gran potencial para ampliar el acceso a la APS, se presenta como una herramienta esencial para la cualificación del trabajo y para el fortalecimiento de la coordinación del cuidado en el SUS.

## Conclusión

En resumen, las actividades de telesalud realizadas por los profesionales de la APS son diversas, integrando actividades relacionadas con las prácticas de gestión, educativas, de investigación y asistenciales, distribuidas en diferentes proporciones en la jornada laboral. Se destacan las actividades que concentraron mayor tiempo de la jornada laboral, tales como consulta, vigilancia en salud, acciones educativas de los trabajadores de salud e intercambio de información sobre cuidados de salud y/o servicios de salud, en su mayoría realizadas mediante llamadas de voz. Además, el estudio presenta patrones de tiempo promedio de las actividades de telesalud, apoyando la discusión sobre el planeamiento y dimensionamiento de la fuerza laboral en salud digital en el contexto de la APS.
